# The raising and westward expansion of central Tibet

**DOI:** 10.1093/nsr/nwaf058

**Published:** 2025-02-21

**Authors:** Chenyuan Zhao, Lin Ding, Zhongyu Xiong, Robert A Spicer, Fulong Cai, Songlin He, Chao Wang, Wenqing Ding, Jinxiang Li, Houqi Wang, Zheng Yin, Xiaoyan Xu, Jing Xie, Yahui Yue, Deng Zeng, Amaneh Kaveh-Firouz

**Affiliations:** State Key Laboratory of Tibetan Plateau Earth System, Environment and Resources (TPESER), Institute of Tibetan Plateau Research, Chinese Academy of Sciences, Beijing 100101, China; University of Chinese Academy of Sciences, Beijing 100049, China; State Key Laboratory of Tibetan Plateau Earth System, Environment and Resources (TPESER), Institute of Tibetan Plateau Research, Chinese Academy of Sciences, Beijing 100101, China; University of Chinese Academy of Sciences, Beijing 100049, China; State Key Laboratory of Tibetan Plateau Earth System, Environment and Resources (TPESER), Institute of Tibetan Plateau Research, Chinese Academy of Sciences, Beijing 100101, China; State Key Laboratory of Tibetan Plateau Earth System, Environment and Resources (TPESER), Institute of Tibetan Plateau Research, Chinese Academy of Sciences, Beijing 100101, China; School of Environment, Earth and Ecosystem Sciences, The Open University, Milton Keynes MK7 6AA, UK; State Key Laboratory of Tibetan Plateau Earth System, Environment and Resources (TPESER), Institute of Tibetan Plateau Research, Chinese Academy of Sciences, Beijing 100101, China; State Key Laboratory of Tibetan Plateau Earth System, Environment and Resources (TPESER), Institute of Tibetan Plateau Research, Chinese Academy of Sciences, Beijing 100101, China; State Key Laboratory of Tibetan Plateau Earth System, Environment and Resources (TPESER), Institute of Tibetan Plateau Research, Chinese Academy of Sciences, Beijing 100101, China; State Key Laboratory of Tibetan Plateau Earth System, Environment and Resources (TPESER), Institute of Tibetan Plateau Research, Chinese Academy of Sciences, Beijing 100101, China; University of Chinese Academy of Sciences, Beijing 100049, China; State Key Laboratory of Tibetan Plateau Earth System, Environment and Resources (TPESER), Institute of Tibetan Plateau Research, Chinese Academy of Sciences, Beijing 100101, China; State Key Laboratory of Tibetan Plateau Earth System, Environment and Resources (TPESER), Institute of Tibetan Plateau Research, Chinese Academy of Sciences, Beijing 100101, China; State Key Laboratory of Tibetan Plateau Earth System, Environment and Resources (TPESER), Institute of Tibetan Plateau Research, Chinese Academy of Sciences, Beijing 100101, China; University of Chinese Academy of Sciences, Beijing 100049, China; State Key Laboratory of Tibetan Plateau Earth System, Environment and Resources (TPESER), Institute of Tibetan Plateau Research, Chinese Academy of Sciences, Beijing 100101, China; University of Chinese Academy of Sciences, Beijing 100049, China; State Key Laboratory of Tibetan Plateau Earth System, Environment and Resources (TPESER), Institute of Tibetan Plateau Research, Chinese Academy of Sciences, Beijing 100101, China; State Key Laboratory of Tibetan Plateau Earth System, Environment and Resources (TPESER), Institute of Tibetan Plateau Research, Chinese Academy of Sciences, Beijing 100101, China; State Key Laboratory of Tibetan Plateau Earth System, Environment and Resources (TPESER), Institute of Tibetan Plateau Research, Chinese Academy of Sciences, Beijing 100101, China; University of Chinese Academy of Sciences, Beijing 100049, China; State Key Laboratory of Tibetan Plateau Earth System, Environment and Resources (TPESER), Institute of Tibetan Plateau Research, Chinese Academy of Sciences, Beijing 100101, China

**Keywords:** paleoelevation, lithospheric delamination, CLAMP, clumped isotope, Tibet

## Abstract

Understanding the Cenozoic growth history of the Himalaya-Tibetan Plateau (HTP) is essential for elucidating the underlying geodynamic mechanism and interactions among topography, biosphere and atmosphere. However, the spatial-temporal evolution of the HTP, especially that of the Paleogene Central Tibetan Valley (CTV), remains hotly debated. In this study, through radiometric geochronology, plant assemblages, oxygen and clumped isotope paleoaltimetries, we reconstruct the uplift history of the east-west-oriented Luolong Basin in eastern Tibet. Results show that the Luolong Basin was at 0.6 (+0.2/−0.4) km at ca. 54–46 Ma, then rose to 2.9 ± 0.9 km at ca. 44 Ma. The newly discovered Luolong Flora indicates the Eocene CTV extending into eastern Tibet, and that the valley was higher in the east, sloping to the west, inferring a westward progressive rise of the valley floor. Integrated evidence from paleomagnetism, magmatism and seismic tomography suggests that the birth of the near modern plateau is attributed to the stepwise delamination (drip) of the subducted Lhasa lithosphere from east to west.

## Teaser

The Eocene Central Tibetan Valley has exhibited a higher elevation in eastern Tibet and gradually descended westward. The progressive delamination of the Lhasa lithosphere from east to west and subsequent asthenosphere upwelling contributed to the demise of this valley system.

## INTRODUCTION

The Himalaya-Tibetan Plateau (HTP) is a complex tectonic collage modified by the north–south ongoing collision of the Indian and Eurasian plates, beginning as early as 65 million years ago [1,2]. Today the plateau has risen to over 4.0 km and the crust has thickened to twice its normal thickness (∼70 km) [3], making it an unusual and ideal place for multi-layer interaction research. The spatial and temporal evolution of the HTP is the result of geodynamic processes of the lithosphere and asthenosphere and continues to play a critical role in regulating the Asian, and even the global, climate system [4,5].

The processes driving the Cenozoic rise of the HTP remain vigorously debated. Early models posited that the HTP underwent a general rise caused by the underthrusting of the Indian plate, or convective removal of the mantle lithosphere [5,6]. However, subsequent evidence indicates that the rise of the HTP was not uniform but multi-staged [7]. The oblique stepwise rise model postulates that as the Indian plate subducted obliquely, ancient sutures were activated resulting in a progressive rise of the HTP from the southwest to the northeast [8]. The lower crustal flow model is expressed as a rapid eastward penetration of lower crustal material from central Tibet into eastern and southeastern Tibet, which is supposed to have contributed to crustal thickening and further uplift in eastern Tibet since ca. 15 Ma [2,9]. Reconstructing plateau development quantitatively and accurately is critical to understanding the geodynamic evolution of the region [10]. However, this remains a significant challenge.

The Paleogene paleo-geomorphology of the HTP has previously been described either as a high and dry ‘proto-Tibetan Plateau’ or comprising ‘two mountain ranges sandwiching a low elevation basin’ [11,12]. Controversies remain for the sedimentary basins along the Bangong-Nujiang Suture Zone (BNSZ) where paleoelevation reconstructions are debated, due in some cases to the lack of an absolute chronological framework and the diversity of paleoaltimetric approaches that have been applied [13,14]. However, credible fossil evidence in the Bangor Basin intimates a low elevation of no more than 2.0 km in the Eocene [15], rather than an elevation of more than 4.5 km persisting since the Eocene [16]. Clumped isotopes combined with wet-bulb temperatures further demonstrate that the elevation of the Lunpola Basin was ∼1.7 km between ca. 50–38 Ma [13]. These different but consilient lines of evidence point to the existence of a lowland in central Tibet, which has been termed the Central Tibetan Valley (CTV). Recent studies suggest that the CTV extended westward to the Gerze Basin [13,17], and encompassed the Lunpola and Nima basins in the middle part of the region, but there is no geological documentation that characterizes and locates the eastern boundary of the CTV to date. Further evidence exploring the bounds of the CTV will help to clarify the Paleocene geomorphic features of the entire HTP.

Eastern Tibet today forms a transition zone between the high-elevation low-relief plateau interior and the lowlands of Southeast Asia, and comprises several lithospheric fragments. Quantifying when and how eastern Tibet formed its present long-wavelength, low-gradient ramp-like topography provides constraints for understanding the evolution and growth of the HTP [18], as well as its influence on biotic evolution and the Asian Monsoon system [19]. Substantial evidence derived from plant fossils and stable isotope approaches suggests that the rise of eastern Tibet began in the middle to late Eocene [20–23], and not, as previously suggested, in the Miocene or Pliocene [24]. To better understand the outward growth and underlying geodynamic mechanisms of the HTP, it is necessary to understand the links between the rise of eastern Tibet and that of central Tibet.

Here, we report the uplift history of the Luolong Basin, situated in eastern Tibet within the BNSZ. We establish the Cenozoic stratigraphic and geochronologic framework through detailed field mapping and U–Pb dating analysis, and then reconstruct the early to middle Eocene paleoelevation and paleoclimate evolution of the basin using clumped isotope thermometry, palynology and numerical analysis of plant fossil assemblages. We find that the Luolong Basin rose from ∼0.6 km in the early Eocene to ∼2.9 km by the middle Eocene. The newly discovered Luolong fossil assemblage shows great diversity (51 fossil leaf morphotypes), and along with the published Jianglang and Dayu floras, indicates that during most of the Eocene, central and eastern Tibet comprised a valley system that was vegetated, at least around lake and river margins. Our evidence depicts a middle Eocene uplift history of eastern Tibet clearly preceding that of central Tibet. Coupled with diachronous eruption of magmatism beginning around 45 Ma in the east and 30 Ma in the west, we infer that the westward progressive rise of the valley floor likely resulted from the diachronous delamination (drip) of the Lhasa lithosphere and subsequent mantle upwelling.

## RESULTS

### Geological setting, stratigraphy and geochronology

**Figure 1.**
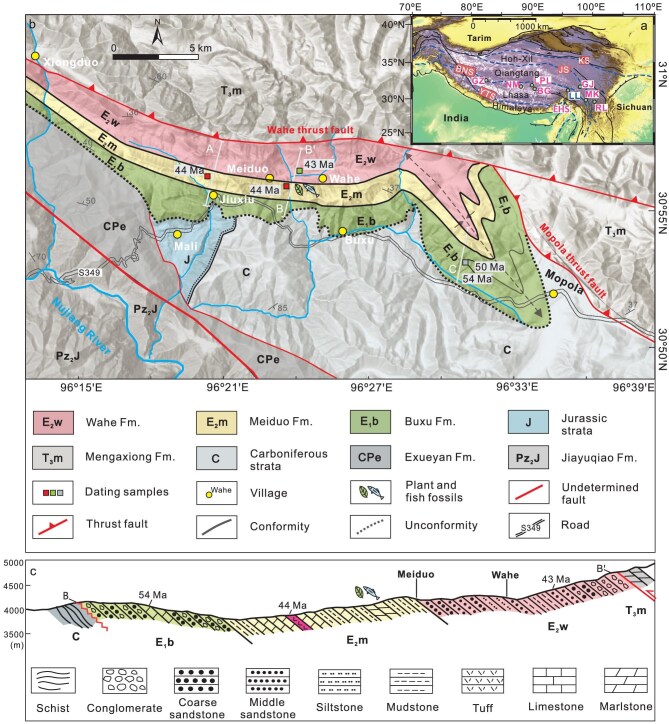
Tectonic context and geological background of the Luolong Basin. (a) Tectonic terranes and sutures of Tibet. From south to north, the major geological entities are: the Himalaya, the Lhasa Terrane, the Qiangtang Terrane and the Hoh-Xil Terrane. The principal sutures are the Yarlung-Tsangpo Suture (YTS), the Bangong-Nujiang Suture (BNS), the Jinshajiang Suture (JS) and the Kunlun Suture (KS). Abbreviations: GZ, Gerze Basin; NM, Nima Basin; LPL, Lunpola Basin; BG, Bangor Basin; LL, Luolong Basin; GJ, Gonjo Basin; MK, Markam Basin; RL, Relu Basin; EHS, Eastern Himalaya Syntaxis. (b) Geological map of the Luolong Basin. The Cenozoic strata include the Buxu Formation (E_1_b), the Meiduo Formation (E_2_m) and the Wahe Formation (E_2_w). A, B and C are three measured sections, and the stratigraphic columns are shown in Fig. S1. (c) Composite geological cross-section of the Luolong Basin showing the main structure and lithology with corresponding age constraints and representative flora. The section position is shown in Fig. 1b as B–B′.

The BNSZ can be traced from the Bangong Lake in the westernmost part, across the Gerze, Dongqiao, Naqu and Dingqing areas, then southeastward along the Nujiang River (Salween River), dividing central Tibet into the Lhasa Terrane to the south and the Qiangtang Terrane to the north [25]. The BNSZ represents the remnants of the Bangong-Nujiang Ocean Basin, the closure of which has accumulated ∼2000 km of shortening since the Early Cretaceous [26,27]. The BNSZ mainly hosts Jurassic flysch, mélange, marine conglomerate and volcanic rocks [27]. The eastern part of the BNSZ converges on the eastern Tibet region, which accommodates significant displacement and crustal deformation from central Tibet [18]. The east-west-oriented Luolong Basin is situated in the northeastern part of the BNSZ, within the middle Nujiang River drainage area (Fig. 1a and b). The Luolong Basin is also within the modern Asian Monsoon transition zone, covering an area of ∼400 km^2^, and encompasses an elevation range of 3600−5200 m. To the north, the boundary of the Luolong Basin is controlled by the Wahe and Mopola thrust systems, which are the secondary thrust faults of the Dingqing Ophiolite Belt. At the southern boundary, Cenozoic strata unconformably overlie Carboniferous sediments (Fig. 1b).

**Figure 2.**
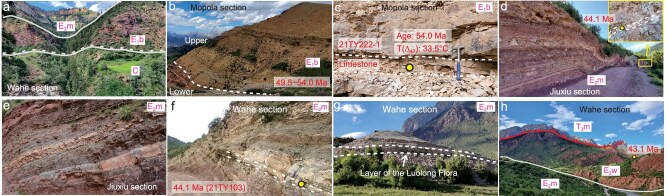
Field pictures of the Luolong Basin. (a) Fluvial coarse sandstone deposition of the lower part of the Buxu Formation, which unconformably overlies Carboniferous metasandstone and phyllite at the Wahe section. (b) The Buxu Formation at the Mopola section could be divided into a lower portion and an upper portion. (c) Limestone layer with corresponding calcite U–Pb dating age and T(Δ_47_) at the upper part of the Buxu Formation, Mopola section. (d) Lacustrine strata within the syn-depositional structure of the Meiduo Formation at the Jiuxiu section. The enlarged picture represents the tuff layer. (e) Lacustrine deposits within diverse-colored mudstone layers of the Meiduo Formation, Jiuxiu section. (f) Tuff layer of the lacustrine strata in the Meiduo Formation, Wahe section. (g) The Luolong Flora in the layer of marlstone of the Meiduo Formation, Wahe section. (h) The fluvial sandstone and conglomerate deposit of the Wahe Formation, which overlies the Meiduo Formation and has been thrusted by Triassic strata, Wahe section.

Comprehensive field work shows that the Cenozoic stratigraphy of the Luolong Basin can be divided into three parts, from the lower to the upper, comprising the Buxu Formation (E_1_b), the Meiduo Formation (E_2_m) and the Wahe Formation (E_2_w) (Figs 1c and 2, Fig. S1).

The Buxu Formation (E_1_b) is an assemblage of alternating purplish to yellow coarse sandstones and white pebbly sandstones interlayered with yellow limestone, marlstone, bioclastic limestone and mudstone in the middle part, with a total thickness of ∼600 m (Fig. 2a–c). This lithological succession intimates an evolutionary pattern of depositional conditions in which water energy started high, subsequently decreased to a moderate level, and eventually resurged again to a high level. The pattern is indicative of fluvio-lacustrine facies. The results of calcite U–Pb dating of two layers of limestone, and zircon U–Pb dating of two sandstone samples, show that the depositional age of the Buxu Formation is constrained to the early Eocene, with an age range from 54 Ma to 46 Ma (Fig. S2a, b, d, e).

**Figure 3.**
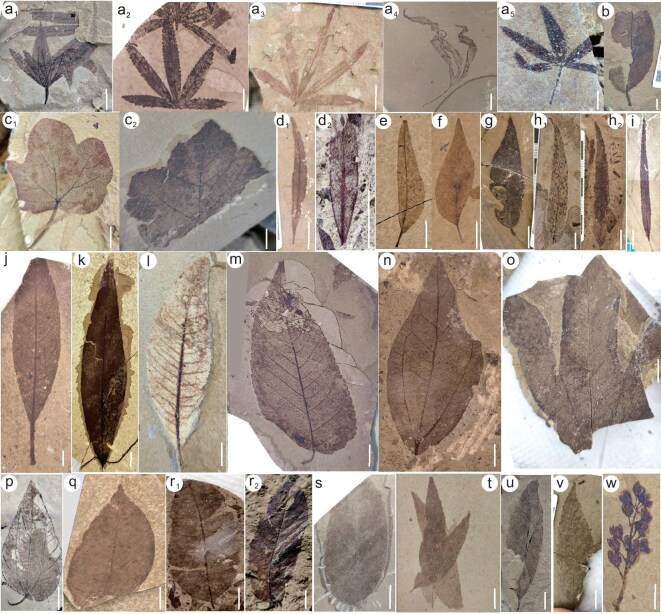
(a–w) Plant assemblages of the middle Eocene Luolong Flora in the Luolong Basin. Scale bars equal to 1 cm. (a_1_–a_5_) *Cannabis* sp., OTU1. (c_1_–c_2_) Malvaceae, OTU41. (d_1_–d_2_) Myrtaceae. (e–g) *Syzygium* sp., e, OTU21, f–g, OTU22. (h_1_–h_2_) *Salix* sp., OTU28. (i) OTU27. (j) *Litsea* sp., OTU8. (k) *Pegia* sp., OTU4. (l) *Terminthia* sp., OTU6. (m) *Zelkova* sp., OTU31. (n) Lauraceae, OTU10. (o) Sterculiaceae, OTU39. (p) *Ziziphus* sp., OTU48. (q) *Populus* sp., OTU16. (r_1_–r_2_) *Quercus* sp.; r_2_, OTU13. (t–u) Overlapping leaves. (v) OTU36. (w) Flowers. OTU is the morphotype shown in Table S6. The leaf fossil in (s) is difficult to identify and was not used for CLAMP analysis.

**Figure 4.**
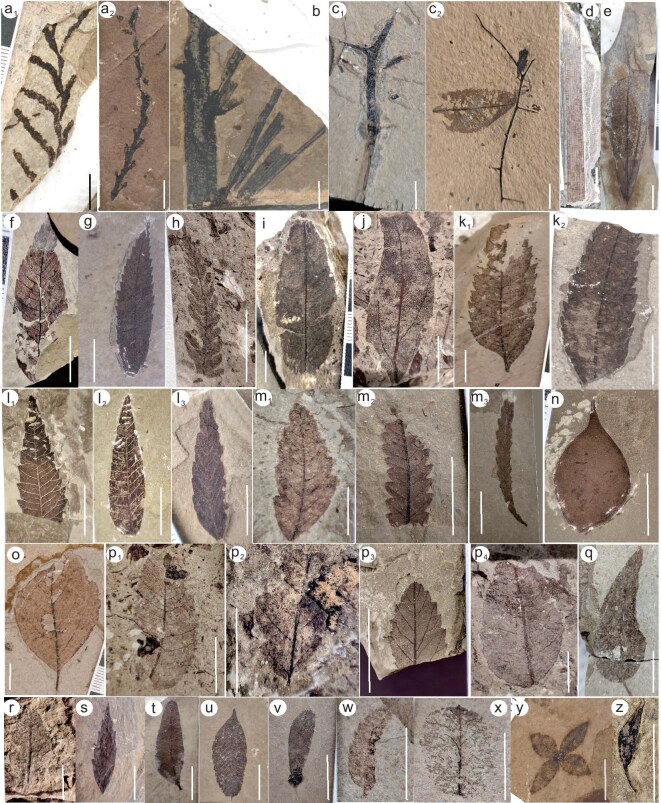
(a–z) Plant assemblages of the middle Eocene Luolong Flora in the Luolong Basin. Scale bars equal to 1 cm. (a_1_–a_2_) Cupressaceae. (b) Palm with pinnate leaf. (c_1_–c_2_) Branch with spines. (d) *Typha* sp. (e) Lauraceae. (f) OTU2. (g) OTU33. (h) Fern. (j) Lauraceae, OTU10. (k_1_–k_2_) *Vauquelinia* sp. (l_1_–l_3_) Anacardiaceae, OTU34. (m_1_–m_3_) *Palibinia* sp., OTU24. (n) *Podocarpium* sp. (o) Magnoliaceae, OTU9. (p_1_–p_4_) *Quercus* sp.; p_1_, OTU12. (q) OTU11. (s) OTU35. (t) *Pistacia* sp. (v) *Ventilago* sp. (x) OTU45. (y) *Forsythia* sp. (z) Bud. OTU is the morphotype shown in Table S6. The leaf fossils (i, r, u, w) are difficult to identify and were not used for CLAMP analysis.

The Meiduo Formation (E_2_m) comprises yellow to green interbedded limestone, marlstone, mudstone and tuff interlayered with purplish red siltstone and fine sandstone, totaling ca. 250 m in thickness. Notably, it preserves algae in the limestone layers and a suite of well-preserved plant fossils (the Luolong Flora), fish and insect remains in the marlstone layer (Figs 2d–g, 3, 4). The Meiduo Formation is thus dominated by lacustrine facies. Zircon U–Pb dating of three tuff layers constrains the depositional time of the Meiduo Formation to 44–43 Ma (Fig. S2f–h).

The Wahe Formation (E_2_w) is an assemblage of purplish red fluvial sediments comprising purplish red medium- to coarse-grained sandstone and conglomerate (Fig. 2h). A sandstone sample obtained at the top of the Wahe section yields three youngest zircon ages with a weighted age of 43.1 ± 1.8 Ma (Fig. S2c), which suggests that the depositional age of the Wahe Formation is no later than 43 Ma.

The evolution of the sedimentary environment of the Luolong Basin can therefore be divided into three stages, which started with the deposition of fluvio-lacustrine facies, then translated into lacustrine facies, and eventually ended with fluvial facies. The sediments within the Luolong Basin accumulated from the early Eocene to the middle Eocene (ca. 54–43 Ma).

### The early Eocene paleoelevation estimate

By combining clumped and oxygen isotope paleoaltimetries, we quantitatively estimate the early Eocene paleoelevation of the Luolong Basin. The essential theory underlying stable isotope paleoaltimetry is the Rayleigh-type distillation process, where stable isotopes deplete systematically with altitude in air parcels ascending against a single mountain barrier [28]. Seasonal changes in the layers of mammalian teeth and ostracods suggest that in the Paleogene, moisture from South Asia influenced the climate of Myanmar and the Gangdese area [12,29]. This suggests that a southerly air flow also dominated the eastern Tibet area during the early Eocene and constituted the primary moisture source for this region. Oxygen isotope records in lacustrine and paleosol deposits are useful materials for deciphering the elevations of the past [30].

**Figure 5.**
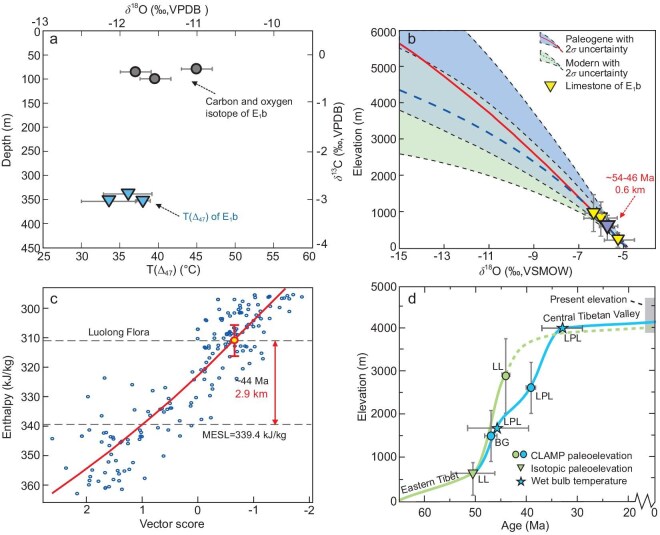
Paleoelevation results for the Luolong Basin. (a) Stable and clumped isotope results from three limestone samples collected from the Buxu Formation. The dots represent the carbon and oxygen isotope results, while the triangles represent the clumped isotope temperature results (T(Δ_47_)) within corresponding sampling depth. (b) Paleoelevation reconstruction through stable isotopic paleoaltimetry of the early Eocene Luolong Basin. (c) CLAMP regression plots showing the middle Eocene paleoelevation of the Luolong Basin. (d) Reconstructed uplift history of the Luolong Basin and the CTV integrating stable isotope paleoaltimetry and CLAMP analysis [13,15,37]. The rise of the Luolong Basin is earlier than the CTV. Abbreviations are as in Fig. 1a. Error bars in Fig. 5a–d represent 1σ.

Three limestone samples (21TY219, 21TY222-1, 21TY222-2) obtained from the Buxu Formation yield Δ_47_ values of 0.651‰, 0.659‰ and 0.646‰, respectively. The temperature estimation protocol proposed by [31] is employed to calculate the temperature due to its inclusion of a large number of sample types and similar testing process, which yield three temperatures of 36.0 ± 3.2°C, 33.5 ± 3.5°C and 37.9 ± 0.8°C, respectively, leading to an average temperature of 35.8 ± 1.8°C (1σ, Fig. 5a). The corresponding δ^18^O values are −11.8‰, −11.5‰ and −11.0‰, respectively (Vienna Pee Dee Belemnite (VPDB), Fig. 5a). Incorporating the clumped isotope temperature (T(Δ_47_)) and fractionation equation [32], the reconstructed oxygen isotope values of paleoenvironmental water (δ^18^O_cw_) are in the range of −7.5‰ to −6.2‰ (Vienna Standard Mean Ocean Water, VSMOW). After accounting for both paleolatitude changes and global cooling effects, the final calibrated δ^18^O_cw_ values are in the range of −6.3‰ to −5.0‰. Using an Eocene empirical formula to estimate paleoelevation [24], the calculated paleo-surface heights are in the range of 50 (+237/−239) m to 947 (+557/−566) m with a mean elevation of 604 (+198/−426) m (Fig. 5b, Table S5). The uncertainties here are the combination of fractionation model and propagated uncertainties (more details of diagenesis assessment and calculating processes are given in the supplementary data).

### The middle Eocene paleoelevation estimate

The Luolong Flora is preserved in a 1.5-m-thick layer of marlstone and mudstone at Meiduo Village, Luolong County, eastern Tibet. The taxa are assigned to the lacustrine sediments of the Meiduo Formation, and so are middle Eocene in age. A total of 2387 fossil specimens were collected including leaves, fruits, flowers, insects and fish (Figs 3, 4, Figs S3–S5). We further divided the leaves into 51 morphotypes of woody dicots and assigned them to more than 16 families encompassing 20 genera.

The middle Eocene paleoelevation reconstruction is made using Climate Leaf Analysis Multivariate Program (CLAMP), which allows us to employ the moist enthalpy (energy conservation) method [33,34]. The PhysgAsia2 data set is selected for calibration due to the location of the fossil flora in Asia. CLAMP analysis based on the PhysgAsia2 data set suggests that the Luolong assemblage records a moist enthalpy of 311.0 ± 8.4 kJ/kg. There is a paucity of paleomagnetic work revealing the position of the north Lhasa Terrane and adjacent BNSZ during the middle Eocene, but one estimate derived from Meisu volcanic rocks, Rutog County, suggests that the southernmost part of the Qiangtang Terrane was at 29.5°N at ca. 40 Ma (33°N today) [35], which records an approximate paleolatitude of the BNSZ. This indicates that the Lhasa Terrane would have been 3° south of its present latitude. The raw sea-level enthalpy value (334 kJ/kg at paleo-location 27.7°N, 96.4°E) is derived from a coupled atmosphere-ocean general circulation model (HadCM3BL-M2.1aD) with Lutetian boundary conditions [20]. However, as model and proxy data are rarely directly comparable, the moist enthalpy at sea level (MESL) needs be further adjusted for systematic differences in model/CLAMP values. An empirical equation for the Eocene is used to adjust the MESL value (y = −0.7167x + 14.4466, where y is the calibrated moist enthalpy value at sea level and x is the paleolatitude) [15]. This yields an adjusted MESL value of 339.4 kJ/kg. Using the principle of energy conservation [34], the reconstructed paleoelevation of the Luolong Flora is 2.9 ± 0.9 km (Fig. 5c). The uncertainty is a combination of those inherent in CLAMP and model/proxy adjustment.

### Paleoclimate reconstruction

In order to reconstruct the early Eocene climate, two grayish-green mudstone samples collected from the upper part of the Buxu Formation, Mopola section, were selected for palynological analysis (Fig. S6). This palynological assemblage is characterized by a high abundance of *Ephedripites* (72.5%). Fern spores are also common (26.5%), represented by *Deltoidospora* (9.8%), *Pterisisporites* (9.3%) and *Polypodiaceaesporites* (7.4%). The early Eocene palynoflora of the Luolong Basin exhibits low taxonomic diversity, and the most conspicuous characteristic is the high percentage of pollen (*Ephedripites*) usually assumed to represent the xerophytic shrub *Ephedra. Ephedra* is a typical desert shrub currently distributed primarily in arid/semi-arid desert regions [36]. The high percentage of *Ephedripites* suggests that the Luolong Basin was dominated by an arid climate, where the landscape was mainly covered by xerophytic shrubs, and that an arid desert ecosystem existed in eastern Tibet during the early Eocene.

The middle Eocene paleoclimate of the Luolong Basin is reconstructed using the plant assemblages and climate parameters inferred from the CLAMP numerical analysis. The representative and recognized plant families and genera comprise tropical-subtropical plants such as members of the Anacardiaceae *(Pegia* sp.*, Terminthia* sp.*, Pistacia* sp.), Hamamelidaceae, Lauraceae *(Litsea* sp.), Myrtaceae *(Syzygium* sp.), palm, and temperate plants including Fagaceae *(Quercus* sp.), Salicaceae *(Populus* sp., *Salix* sp.), Rosaceae *(Vauquelinia* sp.), Sterculiaceae and Ulmaceae *(Zelkova* sp.) families. Malvaceae, Moraceae *(Cannabis* sp.), Magnoliaceae, Leguminosae *(Podocarpium* sp.), Rhamnaceae *(Ziziphus* sp.*, Ventilago* sp.), Oleaceae *(Forsythia* sp.), Cupressaceae, Typhacea *(Typha* sp.) and ferns are adaptable to both subtropical and temperate environments. The extinct *Palibinia* leaves are also preserved. In total, the plant fossil assemblage encompasses over 20 families and 24 genera. The assemblage composition implies a highly diverse subtropical to temperate broad-leaved and deciduous mixed forest (Figs 3, 4).

Climate indices from the CLAMP analysis further strengthen the taxon-based environmental interpretation. The mean annual air temperature (MAAT) is estimated to have been 13.0 ± 2.4°C with an average temperature of 24.9 ± 2.9°C in the warmest month and an average temperature of 2.0 ± 3.5°C in the coldest month. The length of growing season (LGS) is estimated to have been 8.2 ± 1.1 months and the growing season precipitation (GSP) was 1481 ± 643 mm. The seasonal variation range of temperature and the LGS is consistent with a subtropical climate where the minimum temperature is >0°C and the LGS is >8 [33]. The three consecutive wettest months precipitation (X3WET) is estimated to have been 728 ± 400 mm, while the three driest months precipitation (X3DRY) was 217 ± 98 mm. The wet/dry precipitation ratio approximates to 3 : 1, implying a seasonal climate but well short of the seasonality typical of the modern monsoon. The mean annual vapor pressure deficit (VPD. Ann) is estimated to have been 6.9 ± 2.4 hPa. For spring, summer, autumn and winter, the estimated VPD values were 5.0 ± 4.0 hPa, 11.5 ± 3.5 hPa, 8.0 ± 2.0 hPa and 2.8 ± 1.5 hPa, respectively. This means the air was wettest in winter and dryest in summer.

## DISCUSSION

### A Central Tibetan Valley open to the west during the Eocene

During the early Eocene (ca. 54–46 Ma), stable isotope paleoaltimetry coupled with palynological analysis suggests that the Luolong Basin remained at a low elevation of ∼0.6 km and was generally arid. By the middle Eocene (ca. 44 Ma), the Luolong Basin had risen to ∼2.9 km, revealing a ∼2.0 km increase in surface elevation compared to the early Eocene. The Luolong Flora indicates a highly diverse subtropical to temperate broad-leaved and deciduous mixed forest, suggesting that the climate changed to warm and wet. We therefore show a clear uplift history of the Luolong Basin from ∼0.6 km to ∼2.9 km, from the early Eocene to the middle Eocene.

Combining our new results with existing paleoelevation and paleoclimate data, the Eocene evolution of the CTV is revealed. The Jianglang Flora, preserved in the Bangor Basin [15] of central Tibet, and deposited at ca. 47 Ma, suggests great plant diversity, comprising 70 different plant fossil morphotypes, with some species that are now characteristic of lowland floras in southeastern Asia exhibiting their first occurrences in central Tibet. Notable taxa include members of the Apocynaceae, *Ceratophyllum, Illigera, Lagokarpos* and Vitaceae [15]. CLAMP analysis reveals that the basin was at an elevation of 1.5 ± 0.9 km. Furthermore, clumped isotope and wet-bulb temperature lapse rate analyses also demonstrated that the central Tibet region was low (1.7 +0.9/−0.8 km) [13]. The late Eocene Dayu Flora preserved in the Lunpola Basin, 50 km north of the Jianglang Flora in central Tibet, was at an elevation of 2.6 ± 0.9 km, but exhibits a markedly lower biodiversity than the Jianglang Flora, with palm, *Ailanthus, Cedrelospermum, Koelreuteria* and *Limnobiophyllum* being common [37]. The vegetation is reconstructed to have been more open subtropical woodland [37]. Comparison of the Jianglang Flora with the Dayu Flora indicates that the vegetation of the central Tibet region transformed from a closed forest at low-elevation to a moderate-elevated open woodland [33,37]. Further west along the BNSZ, near-contemporary foraminifera fossils are preserved in the Gerze Basin indicating a lower elevation [17].

Our plant fossil evidence and CLAMP analysis suggest that the Luolong Basin was at ∼2.9 km during the middle Eocene. The vegetation, including species belonging to Anacardiaceae, Lauraceae, Myrtaceae, Leguminosae, Rosaceae and Hamamelidaceae, shows a similarity to that represented by the Jianglang and Dayu fossil floras, extending the subtropical ecosystem into eastern Tibet. Further to the southeast in the SE Tibet area, a subtropical to temperate evergreen and deciduous broadleaf mixed forest was preserved in the Markam and Relu basins, as evidenced by the MK-DR, MK3, MK1, TR1 and TR3 floras [20,21,23]. Our evidence suggests that the Eocene Luolong Basin occupied a pivotal geological position that connected central and eastern Tibet, and unified a lowland subtropical forest zone along the BNSZ then extended into eastern Tibet. During the middle Eocene, the Gonjo Basin had reached an elevation of 3.8 km [22], while the Markam and Relu basins were at a moderate elevation of 2.5–2.9 km [20,23]. In conjunction with the elevation of the Luolong Basin, which stood at 2.9 km, it can be surmised that eastern Tibet attainted a measurably higher elevation than central Tibet during the middle Eocene (Fig. 5d). Low-temperature thermochronological evidence from the Rutog area in western Tibet also suggest the development of a westward drainage system [38]. Therefore, the topography of the CTV was characterized by a westward-sloping configuration, beginning higher in the east and gradually lowering to the west.

### Lithospheric delamination drove the westward expansion of central Tibet

This paleo-geomorphic scenario for the CTV, in which the valley floor was much higher in the east and lower in the west, indicates that the surface uplift of eastern Tibet occurred before that of central Tibet. Existing paleoelevation studies suggest that eastern Tibet rose substantially in the middle to late Eocene to reach near-present elevation [20–23]. In contrast, the rise of the western CTV occurred later than that of eastern Tibet, terminating with the deposition of the Dingqinghu Formation between 40 Ma and 20 Ma [13,33]. Considering previous metamorphic events, thrust fault activity, paleomagnetic evidence, igneous activity and seismic tomography, we propose a new mechanism underpinning this surface uplift process.

The Eastern Himalaya Syntaxis (EHS) is located at the frontal part of the eastern termination of the Himalayan collisional orogen. The medium- to high-grade metamorphism of amphibolite- and granulite-facies, dating to ca. 65–40 Ma, indicates that northeastern greater India penetrated deeply beneath the Eurasian continent [39–41]. This process resulted in the subduction of crustal materials to depths exceeding 70 km along with extensive tectonic deformation and crustal shortening [40,41]. The strong indentation of the Indian plate would likely transfer to the eastern part of the Lhasa Terrane, and resulted in more intense intracontinental subduction between the Lhasa and Qiangtang terranes. However, the ongoing intracontinental subduction would have been hampered by increasing buoyancy and eclogitization of the lower crust and mantle lithosphere, leading to lithospheric tearing and break-off [42–44]. Seismic tomography imaging reveals the presence of a high-velocity anomaly zone in central Tibet at over ∼100−400 km depth, which probably represents the remnants of the dripping Lhasa lithosphere [45]. Another clear high-velocity anomaly is detected under eastern Tibet [46,47], indicating that the delamination (drip) of the Lhasa lithosphere is a widespread phenomenon. Additionally, recent paleomagnetic investigations reveal that central to western Tibet experienced a latitudinal convergence of >1000 km during 67–30 Ma [35]. Whereas eastern Tibet experienced 200−1000 km latitudinal convergence, 25°−45° counterclockwise rotation and 25°−50° clockwise rotation during the Eocene [48–50]. Substantial convergence indicates significant northward subduction, with the subsequent gravity instability promoting subducted lithospheric mantle tearing and delamination (drip). The time of progressive westward delamination (drip) of the lithosphere is constrained by Eocene–Oligocene calc-alkaline volcanism, which began in eastern Tibet (45–34 Ma) and later occurred in central Tibet (38–27 Ma) [42,44,51]. The subsequent upwelling of asthenosphere materials and magmatic underplating therefore contributed to the topographic uplift.

**Figure 6.**
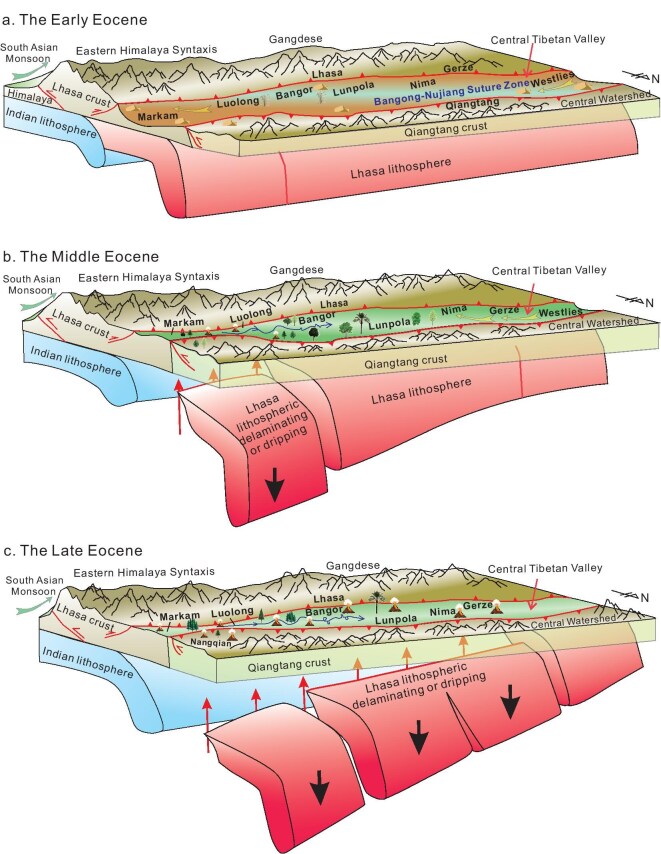
Schematic models illustrating the Eocene westward uplift of the CTV. (a) Since the early Eocene, the Lhasa lithosphere has subducted beneath both the CTV and the Qiangtang Terrane, with the elevations of both central Tibet and eastern Tibet being approximately 1.0 km. (b) In the middle Eocene, the strong indentation of the Indian continent and the increasing buoyancy and eclogitization of the mantle lithosphere culminated in the easternmost segment of the Lhasa lithospheric mantle tearing and breaking off. Asthenosphere upwelling and volcanism resulted in the topographic rise of the eastern Tibet area. (c) During the late Eocene, the Lhasa lithosphere underwent a secondary stage of delamination (drip) further westward along central Tibet leading to the rise of the CTV floor and nascent plateau formation.

The westward progressive rise of CTV and its underpinning mechanism can be summarized as follows: during the Paleocene to the early Eocene, the oblique subduction of the Indian plate drove the Lhasa lithosphere northward and northeastward, leading to the formation of a thickened lithospheric mantle. This tectonic process triggered large-scale shortening, convergence and clockwise rotation in eastern Tibet. As a result, the Lhasa mantle lithosphere underwent tearing in areas where the most intense deformation occurred (Fig. 6a). Following this rifting, the eastern Lhasa lithosphere experienced its first episode of delamination (drip). The upwelling of asthenosphere material subsequently induced the uplift of eastern Tibet during the middle Eocene (Fig. 6b). Thereafter, the delamination progressively shifted westward, causing the stepwise uplift of the CTV (Fig. 6c).

However, the Luolong Basin did not reach its present elevation (∼4.5 km) in the middle Eocene (∼2.9 km) (Fig. 5d). When integrated with published paleoelevation work in the adjacent Gonjo, Markam and Relu basins [20,22,23], the later episode of uplift of the Luolong area is assumed to coincide with the regional rise of eastern Tibet, which was mainly in the late Eocene. As a result, a high-altitude plateau topography finally formed at the end of the Oligocene, produced by the westward progressive rise of central Tibet driven by the removal of the Lhasa lithosphere. The northern boundary of this early plateau was along the Central Watershed (Tanghula Mountains), stretching south to the Gangdese Mountains, proceeding westward into the Ngari area, and extending east to where the highly dissected Hengduan Mountains are now.

### Green belt/oasis in the Eocene Tibet

During the early Eocene, the ‘red beds’ of the Buxu Formation in the Luolong Basin show characteristics of fluvial-lacustrine facies. The T(Δ_47_) indicates that the limestones were precipitated at a relatively high temperature of ∼35.8°C. Palynological analysis reveals that an *Ephedra*-like plant contributed greatly to the pollen spectrum (∼70%), suggesting a relatively arid environment supporting mostly xerophytic shrubs. Both the T(Δ_47_) and the vegetation point to a hot and arid climate. Despite the absence of classic aeolian dune deposits in the Buxu Formation, the low elevation and arid climate suggest close similarities with the pervasive distribution of Cretaceous–early Paleogene aeolian deposits from eastern Tibet to southern China [52].

After 44 Ma, the Luolong Basin witnessed the deposition of grayish-green lacustrine sediments, implying a climate transformed from arid to semi-humid. The Luolong Flora represents a highly diverse subtropical to temperate broad-leaved and deciduous mixed forest. This indicates that the extensive forest type observed in the CTV also extended into eastern Tibet. In this broad valley system, the VPD values of the Dayu Flora, the Jianglang Flora, the Luolong Flora, Markam floras (MK-DR, MK3, MK1) and Relu floras (TR1, TR3) show similar characteristics, being at a minimum in winter and maximum in summer [15,20,23,37]. Therefore, the climate in the valley could be described as having been cool and wet in winter and warm and dry in summer, which contrasts markedly with the present monsoon climate in central and eastern Tibet. The plant fossils, like those of palm and *Palibinia* leaves in the Luolong Basin and palm leaves in the Lunpola Basin, are representative species of paleo-Mediterranean-type floras [53]. It seems that during the middle Eocene the climate in parts of the CTV was comparable to that of eastern Tibet, as evidenced by the Markam and Relu basins, which were subject to a distinctive Mediterranean-like climate [23]. Such a summer-dry and winter-wet climate pattern differs from the monsoonal climate in the Yunnan region, which was primarily driven by the seasonal migration of the Intertropical Convergence Zone (ITCZ) [54]. The Mediterranean-like climate would transform to a near-modern Asian monsoon only with the rise of eastern Tibet and subsequent demise of the CTV to form an early version of the modern plateau. What is intriguing is that the rise of eastern Tibet and variation in climate in the middle–late Eocene led to the beginning of a biodiversity hotspot within the Hengduan Mountains. Later, the rise of central Tibet resulted in a shift to much cooler climate conditions supporting high-elevated mixed coniferous-broadleaved forests to alpine steppes as seen today [55].

## CONCLUSIONS

Clarifying the Cenozoic topographic evolution of the eastern Tibet region is of great importance, as it connects the geodynamic mechanisms of outward plateau growth with the evolution of the Asian Monsoon system, large rivers, chemical weathering and biodiversity. Sedimentary analysis and U–Pb geochronology constrain the deposition of Cenozoic strata in the Luolong Basin to the early to middle Eocene, with ages ranging from 54 Ma to 43 Ma. Clumped isotope thermometry coupled with palynological analysis and oxygen isotope data of lacustrine carbonates suggest that during the early Eocene the Luolong Basin experienced an arid desert climate at low elevation. Newly discovered leaf fossil assemblages show that the basin had risen to an elevation of ∼2.9 km by ca. 44 Ma. The diverse vegetation indicates that during the middle Eocene the Luolong Basin was connected to both central Tibet and eastern Tibet, along the foothills of surrounding mountain chains and within a long and wide valley system. Our evidence depicts a middle Eocene uplift history of eastern Tibet that predates the surface uplift of central Tibet. The middle Eocene rise of the Luolong Basin was likely caused by the stepwise delaminating or dripping of Lhasa lithosphere, accompanied by asthenosphere convection and upwelling, contributing to the elevation gain.

## MATERIALS AND METHODS

### U–Pb geochronology

Six tuff and sandstone samples were collected systematically, and zircons were extracted from them. Zircon U–Pb dating was performed using an ESL NWR193UC Excimer laser coupled with an Agilent 7500a inductively coupled plasma mass spectrometer (LA-ICP-MS). Ablation depth was 30 µm with a repetition rate of 6 Hz, and ages were calibrated with Plešovice and 91 500 standard zircons. Two limestone samples were analyzed for calcite U–Pb dating with an Agilent 7900 ICP-MS and an ESL NWR193HE Excimer laser. Calibration utilized NIST SRM 614 and ARM-3, and data correction was done with WC-1 and TARIM standards. All analyses were performed at the State Key Laboratory of Tibetan Plateau Earth System, Environment and Resources (TPESER), Institute of Tibetan Plateau Research, Chinese Academy of Sciences (ITPCAS). Full results are provided in Tables S1 and S2.

### Clumped and stable isotope analyses

Clumped isotope (Δ_47_) analyses were performed at TPESER through a manual vacuum line for CO_2_ extraction and purification. Carbonate samples reacted with phosphoric acid at 90°C, with CO_2_ purified through LN2 traps and a PoraPak Q trap. Δ_47_ was measured using a MAT 253 Plus isotope ratio mass spectrometry (IRMS), corrected for pressure baseline drift and standardized to the Absolute Reference Frame [56]. Data were processed with ‘Easotope’. Carbon and oxygen isotope analyses were conducted at the Laboratory for Stable Isotope Geochemistry, Institute of Geology and Geophysics, Chinese Academy of Sciences (IGGCAS) using a GasBench Ⅱ and MAT 253 IRMS, achieving a precision of <0.2‰. Results are presented in Tables S3 and S4.

### Fossil material preparation and CLAMP analysis

A total of 2387 fossil specimens including 1996 plant fossil specimens were collected from grayish-green marlstone and mudstone layers at Meiduo Village, Luolong County. The fossils were cleaned, numbered, photographed with a Canon R5 camera, and curated at TPESER, ITPCAS. Identification was based on comparisons with modern plant architecture and published materials. Leaf physiognomy was scored following CLAMP protocols, and paleoclimate signals were calibrated using PhysgAsia2 paired with WorldClim2 data sets [57]. Elevation (Z) was calculated by subtracting moist enthalpy at the fossil site from contemporaneous sea-level value. All data are available in Tables S6–S8.

Moist static energy (h) is conserved as a parcel of air rises, and is the sum of moist enthalpy (H) and potential energy (gZ, where g is gravitational acceleration with a value of 9.81 cm/s^2^, and Z is height) [34]. The elevation (Z) is calculated by subtracting the moist enthalpy at the fossil site (*H*_fossil site_) from that at sea level at the same latitude (*H*_sea level_) and dividing by the acceleration due to gravity (g):


\begin{eqnarray*}
{\mathrm{Z}} = \left( {{H_{{\mathrm{sea\, level\,\,}}}} - {H_{{\mathrm{fossil\, site}}}}} \right)/{\mathrm{g}}.
\end{eqnarray*}

### Palynological analysis

Two samples were collected for palynological analysis from the layers of grayish-green mudstone within the Buxu Formation, Mopola section. After chemical pretreatment, the residues were ultrasonically processed and preserved in glycerin. Palynomorphs were mounted on slides and identified under 400 × magnification, with 201 and 221 palynomorphs counted per sample. All the pretreatment and identification work was performed at TPESER, ITPCAS.

## Supplementary Material

nwaf058_Supplemental_File
