# Supplementary material for: The raising and westward expansion of central Tibet
Source: Natl Sci Rev. 2025 Feb 21;12(5):nwaf058. doi: 10.1093/nsr/nwaf058 (PMC12010958; doi:10.1093/nsr/nwaf058)
Supplement: nwaf058_Supplemental_File [file nwaf058_supplemental_file.pdf]

Supplementary Materials for

The raising and westward expansion of central Tibet

Chenyuan Zhao[1,2], Lin Ding[1,2,*], Zhongyu Xiong[1], Robert A. Spicer[1,3], Fulong Cai[1], Songlin He[1], Chao Wang[1], Wenqing Ding[1,2], Jinxiang Li[1], Houqi Wang[1], Zheng Yin[1,2], Xiaoyan Xu[1,2], Jing Xie[1], Yahui Yue[1], Deng Zeng[1,2], Amaneh Kaveh-Firouz[1]

[1]State Key Laboratory of Tibetan Plateau Earth System, Environment and Resources (TPESER), Institute of Tibetan Plateau Research, Chinese Academy of Sciences, Beijing 100101, China

[2]University of Chinese Academy of Sciences, Beijing 100049, China

[3]School of Environment, Earth and Ecosystem Sciences, The Open University, Milton Keynes MK7 6AA, UK

*Corresponding author. E-mail address: dinglin@itpcas.ac.cn (Lin Ding)

This file includes:

1. Stratigraphy and geochronology framework

2. Materials and methods

3. Diagenesis assessment

4. The early Eocene paleoelevation estimate

5. Figures S1 to S7

Figure S1 Three stratigraphic columns in the Luolong Basin including the Jiuxiu section, the Wahe section and the Mopola section

Figure S2 U−Pb dating results of the Luolong Basin

Figure S3 Plant fossils of the middle Eocene Luolong Flora in the Luolong Basin

Figure S4 Plant fossils of the middle Eocene Luolong Flora in the Luolong Basin

Figure S5 Fish and insect fossils from the same layer of the Luolong Flora in the Luolong Basin.

Figure S6 Photographs of pollen grains from the Buxu Formation in the Luolong Basin.

Figure S7 Petrographic images of the limestone samples from the Buxu Formation, Mopola section, Luolong Basin.

6. Tables S1 to S8

Table S1 Zircon LA-ICP-MS U–Pb in-situ results

Table S2 Calcite LA-ICP-MS U–Pb in-situ results

Table S3 Clumped isotopic results of three limestone samples

Table S4 Stable isotopic results of three limestone samples

Table S5 Stable isotopic paleoelevation reconstruction

Table S6 Climate Leaf Analysis Multivariate Program (CLAMP) scoresheet for the Luolong Flora

Table S7 CLAMP scores of leaf fossil physiognomy in the Luolong Flora

Table S8 Climate parameters of the Luolong Flora

7. References

1. Stratigraphy and geochronology framework

Three sections were measured in the western, central and eastern parts of the Luolong Basin, named as the Jiuxiu, Wahe, and Mopola sections, respectively. The detailed lithological characteristics are provided below (Fig. 2 and Fig. S1).

The Buxu Formation (E_1b) unconformably overlies Carboniferous metasandstone and phyllite (Fig. 2a) with a total thickness of ~600 m. The complete Buxu Formation is exposed at the Mopola section and can be divided into two parts based on lithological characteristics (Fig. 2b). The Jiuxiu section and Wahe section only contain the lower portion of the Buxu Formation. In the Mopola section, the lower portion of the Buxu Formation consists of alternating layers of purplish coarse sandstone and white pebbly sandstone, with a total thickness of ~280 m. The layers exhibit thick bedding, and the coarse sandstone shows climbing ripple cross-lamination. The alternating deposition of two different lithologies indicates high water energy within multistage channels. However, the lower portion of the Buxu Formation is 150-m-thick purplish red coarse sandstone in both the Wahe and Jiuxiu sections. Moreover, the lithology in the Jiuxiu section exhibits a gradual change, evolving into an assemblage of purplish coarse sandstone with gravel, fine- to middle-grained sandstone and bedded mudstone. This indicates that the water energy became relatively moderate. Therefore, the lower portion of the Buxu Formation represents a fluvial environment characterized by relatively high water energy. The upper part of the Buxu Formation comprises an assemblage of ~120-m-thick interbedded yellow limestone, marlstone, bioclastic limestone, and layers of grey mudstone (Fig. 2c). The layer of bioclastic limestone contains preserved fossils of snails, ostracods, and algae, indicating a transformation of the sedimentary environment into lacustrine deposition. The uppermost part of the Buxu Formation consists of approximately 200 m of yellow to purplish coarse sandstone with gravel, suggesting a re-increase in water energy. Overall, the sedimentary environment of the Buxu Formation can be defined as fluvial-lacustrine facies. Two sandstone samples (21TY231, 21TY281) collected from the lowest part of the Jiuxiu section and the Mopola section are used to constrain the maximum depositional age of the Buxu Formation. The three youngest zircons from sample 21TY231 yield a weighted mean age of 46.5 ± 1.0 Ma (Fig. S2a), whereas the two youngest zircons from sample

21TY281 represent 47.3 ± 1.0 Ma (Fig. S2b). In addition, two limestone samples (21TY222, 22TL025) were collected from the upper part of the Mopola section. The Tera-Wasserburg Concordia diagrams yield lower intercept ages of 54.0 ± 11.0 Ma (1σ, n = 98, MSWD = 1.4) and 49.5 ± 5.5 Ma (1σ, n = 99, MSWD = 1.6), respectively (Figs. S2d and S2e). Consequently, the depositional age of the Buxu Formation can be constrained to the early Eocene, ranging from 54 Ma to 46 Ma.

The complete Meiduo Formation (E_2m) is exposed at the Wahe section, with a thickness of ~250 m, and comprises yellow to green interbedded limestones, marlstones, mudstones, and tuffs interlayered with purplish red siltstone and fine sandstones (Fig. 2d−f). Algae are preserved in the limestone layers. The upper layer of marlstone also contains a considerable number of plant and fish fossils, and the plants being collectively referred to as the Luolong Flora (Fig. 2g). At the Jiuxiu section, the total thickness of the Meiduo Formation is ~300 m. The layers of purplish-red siltstone and fine sandstone have been deformed into broad, gentle folds and are interlayered with tuffs (Fig. 2d). Given the characteristics of lithology and fossils, the Meiduo Formation is more accurately described as lacustrine deposition. Two tuff samples (22TL041, 21TY125) collected from the Meiduo Formation at the Jiuxiu section yield Concordia ages of 44.1 ± 0.2 Ma (1σ, n = 43) and 43.7 ± 0.6 Ma (1σ, n = 23), respectively (Figs. S2f and S2g). Another tuff sample (21TY103) collected from the Meiduo Formation at the Wahe section, yields a Concordia age of 44.1 ± 0.5 Ma (1σ, n = 17) (Fig. S2h). Three concordant ages suggest that the depositional age of the Meiduo Formation is ~44−43 Ma.

The Wahe Formation (E_2w) is composed of purplish-red fluvial sediments, with a thickness of ~120 m in the Jiuxiu section, and ranging from 180 to 550 m in the Wahe section (Fig. 2h). This formation consists of three lithographic units. The Jiuxiu section records the lowermost part of the Wahe Formation, characterized by alternating layers of bedded mudstone and medium-grained sandstone. The middle and upper parts of the Wahe Formation are only deposited in the Wahe section. The middle part is distinguished by purplish-red medium- to coarse-grained sandstone, occasionally containing gravel. Various sedimentary structures are observed, such as oblique bedding, convolute bedding, and load casts. The upper part consists of a 200-m-thick conglomerate. The gravels are composed of Mesozoic sandstone and limestone. Additionally, there is a relatively low abundance of mudstone, phyllite and quartzite. One sandstone sample (21TY265) obtained from the top of the Wahe

section yields three youngest zircons with a weighted age of 43.1 ± 1.8 Ma (Fig. S2c), suggesting that the depositional age of the Wahe Formation was no later than ~43 Ma.

In summary, the sedimentary environment of the Luolong Basin can be divided into three stages from the bottom to the top: starting with the fluvial-lacustrine facies deposition, transitioning into lacustrine facies, and eventually ending with fluvial facies. The Paleogene deposits in the Luolong Basin span the early Eocene to the middle Eocene (~54−43 Ma).

2. Materials and methods

2.1 U−Pb geochronology

Zircons were extracted using routine crushing and heavy mineral separation techniques. The extracted zircons were mounted in epoxy and polished to expose grain interiors. Cathodoluminescence (CL) imaging was used to characterize the potential complexities with JEOL JSM-IT300. Zircon U−Pb dating was performed using an ESL NWR193UC Excimer laser (primarily NewWave Instrument, USA) coupled to an Agilent 7500a inductively coupled plasma mass spectrometer (LA-ICP-MS). The CL images and dating work were performed at the State Key Laboratory of Tibetan Plateau Earth System, Environment and Resources (TPESER), Institute of Tibetan Plateau Research, Chinese Academy of Sciences (ITPCAS). The ablation of zircon was conducted at a depth of 30 μm, employing a repetition rate of 6 Hz and delivering an energy input of approximately 3 J/cm². Plešovice standard zircons (337 ± 0.37 Ma) was used as an external standard for matrix calibration, and 91500 standard zircon (1064 ± 4.0 Ma) was used to monitor the reliability of the experiment [1, 2]. External calibration of trace element content utilized the NIST SRM 610 standard glass, with ^{29}Si serving as the internal standard element. Offline calculations of isotope ratios and trace element concentrations were conducted using Iolite V4 software, with ordinary Pb correction applied [3]. Ages with an uncertainly exceeding 10% were not included. The U−Pb dating results of the tuff samples were placed on the Wetherill-type Concordia diagram within 1σ. The dating results of the sandstone were presented with probability density, with the three youngest zircon ages being used to constrain the age within 2σ. All zircon U−Pb data are provided in Table S1.

Two limestone samples were collected for calcite U−Pb dating analysis. The in-situ U−Pb dating of calcite was accomplished in the TPESER, which is equipped with an Agilent 7900 Q-ICP-MS coupled with an ESI NWR193HE 193nm ArF excimer laser ablation system. This system is characterized by a short pulse width (20 ns), continuous variable spot sizes (1−300 μm) and a TV3 two-volume sample chamber. The sample-ablated aerosols from the laser were carried by high-purity helium gas (550−650 mL/min flow rate) and 3 mL/min increasing sensitivity-used nitrogen gas, controlled by the mass flow controller in the laser system. Whereafter, the mixed aerosols carried by helium and nitrogen were mixed with the argon gas (0.97−1.02 L/min) from the ICP-MS and entered into the ICP-MS system. The conditions for laser ablation included a spot size of 130−150 μm, an energy density of approximately 3 J/cm^2, and a repetition rate of 10 Hz. At the onset of each analysis, a pre-ablation of 1 second and a background accumulation of 15 seconds were performed, followed by a 20-second sample ablation and a subsequent 5-second washout phase. The two glass reference materials NIST SRM 614 and ARM-3 were employed [4, 5]. One popular reference material WC-1 was used as a primary external standard for the calibration of U/Pb ratios [6]. The TARIM (208.5 ± 0.6 Ma) standard was analyzed as a secondary standard to monitor data accuracy [7]. The instrument drift, isotope fractionation and common Pb corrections were accomplished by using the two-step method within 1σ [7]. Related data are available in Table S2.

2.2 Clumped and stable isotope analyses

Clumped isotope (Δ_{47}) analyses were conducted at TPESER. The extraction and purification of CO_2 was performed on a customized manual vacuum line. Each carbonate sample (6−8 mg) underwent initial reaction with approximately 1 ml anhydrous phosphoric acid at 90°C for 15 minutes to liberate CO_2. The produced CO_2 underwent purification through three liquid nitrogen (LN2) traps (−196°C) and one PoraPak Q trap maintained at −15°C to eliminate moisture and organic contaminants. Analysis of Δ_{47} in purified CO_2 samples was conducted using a MAT 253 Plus dual-inlet isotope ratio mass spectrometer (IRMS), which was set up to measure mass 44−49 CO_2. Correction of pressure baseline drift in sensitive collectors (m/z 47, 48, and 49) was facilitated by configuring an additional detector at mass 47.5. Raw Δ_{47} values were standardized to the Absolute Reference Frame using data from

1000°C heated gas and 25°C equilibrated gas [8]. Acid digestion offsets were corrected using a standard sample transfer function (STF) [9]. Data processing was completed using the "Easotope" software [10]. Clumped isotope data are presented in Table S3.

Carbon and oxygen isotope analyses were conducted using a Finnigan MAT 253 Isotope Ratio Mass Spectrometer with a GasBench II at the Laboratory for Stable Isotope Geochemistry, Institute of Geology and Geophysics, Chinese Academy of Sciences (IGGCAS). Orthophosphoric acid (specific gravity = 1.85 g/cm^3) was used to react with the samples at 72°C in the GasBench II under helium gas flushing conditions. Subsequently, the generated CO_2 was transferred to the MAT 253. The National reference materials GBW4405 and GBW4416 were analyzed repeatedly, demonstrating a laboratory precision better than 0.2‰ for carbon and oxygen isotopes. The isotope results were presented using the standard delta (δ) notation with respect to the Vienna Pee Dee Belemnite (VPDB), and were represented as $\delta^{18}O_c$ and $\delta^{13}C_c$ in per mil (‰). All data are available as Table S4.

2.3 Fossil materials preparation and CLAMP analysis

A total of 2,387 fossil specimens were collected at Meiduo Village, Luolong County. The fossils are preserved in layers of greyish-green marlstone and mudstone. All the fossils were cleaned and numbered in the field, after which they were photographed and curated in the TPESER. The images were captured using an artificial light source with a Canon R5 camera. A macro-lens was used to record the microstructure of the leaf. The fossils were identified primarily through comparison of their architectural features with those of modern plants, combined with a review of published fossil materials. Subsequently, the protocols outlined on the CLAMP website (http://clamp.ibcas.ac.cn) were followed, with a numerical description and scoring of leaf physiognomic characteristics conducted. The paleoclimate signal preserved in the fossil leaf assemblages was derived using the PhysgAsia2 physiognomic calibration file paired with 1-km resolution gridded climate dataset, WorldClim2, to calibrate CLAMP [11]. The relevant data are provided in Tables S6−S8.

Moist static energy (h) is conserved as a parcel of air rises, which is the sum of moist enthalpy (H) and potential energy (gZ, where g is gravitational acceleration with a value of 9.81 m/s^2, and Z is height) [12]. The elevation (Z) is calculated by subtracting the moist enthalpy at the fossil site (H_{fossil}

$_{site}$) from that at sea level at the same latitude ($H_{sea\ level}$) and dividing by the gravitational constant (g):

$$Z = (H_{sea\ level} - H_{fossil\ site}) / g$$

2.4 Palynological analysis

Two samples were collected for palynological analysis from a layer of greyish-green mudstone in the Buxu Formation, Mopola section. All the pretreatment and identification work were performed at the TPESER. Approximately 30 g of the crusted samples were successively treated with 10% HCl and 40% HF to remove carbonates and silicates. Then the residues were washed repeatedly with ultrapure water until the liquid was neutralized. After three minutes of ultrasonic concussion and centrifugation, the palynomorphs were transferred to 2 ml test tubes and glycerin was added for preservation. The extracted samples were mounted on glass slides and identified under an optical microscope with 400× magnification. A total of 201 and 221 palynomorphs were identified and counted for each sample.

3. Diagenesis assessment

Only primary carbonate isotopic values can be used for paleoelevation determination. Here, we combine petrographic analysis and clumped isotope temperature to evaluate potential diagenetic effects on the limestone samples from the Mopola section.

Thin sections of these limestone samples predominantly show a micritic matrix (Fig. S7). There is only sparse sparry fabric or filled veins observed in the thin sections. The petrographic analysis suggests a high probability that the carbonate samples have not undergone discernible recrystallization.

Clumped isotope temperature is a more robust indicator for assessing potential pedogenic processes, such as solid-state reordering of C−O bonds [13]. Three limestone samples (21TY219, 21TY222-1, and 21TY222-2) from the Mopola section yield Δ_{47} values of 0.651‰, 0.659‰ and 0.646‰, corresponding to calculated temperatures of 36.0°C, 33.5°C and 37.9°C. The temperature range is lower than the threshold commonly defined as altered, which is typically 40°C in lower-middle latitude areas [14]. This is also significantly lower than the temperatures associated with solid-state reordering (~100°C) [13]. Therefore, both petrographic analysis and clumped isotope temperatures

suggest that the limestone samples retain their primary isotopic information.

4. The early Eocene paleoelevation estimate

Prior to attempting any elevation estimates, it is necessary to apply corrections for palaeolatitude and global cooling effects [15, 16]. Existing paleomagnetic data suggests that the Lhasa Terrane has moved northward by ~10° since the early Eocene [17]. This would result in an underestimate value of −0.8‰ of the oxygen isotope values of paleo-environmental water ($\delta^{18}O_{cw}$) [18]. In consideration of global cooling since the early Eocene, this would result in a correction of +2.0‰ [19]. After accounting for both paleomagnetic effects and global cooling effects, the final correction to the paleo-environment water ($\delta^{18}O_{cw}$) is +1.2‰ and the final calibrated values of $\delta^{18}O_{cw}$ are in the range of −6.3‰ to −5.0‰ (VSMOW). Using an empirical formula to estimate the paleoelevation [20], the mean calculated paleo-surface elevation is 604 (+198/−426) m (Fig. 5b, TableS5). The uncertainties here are those arising from the combination of the fractionation model and propagated uncertainties.

Additionally, we employ clumped isotope temperatures to further estimate the paleoelevation. The global mean sea surface temperature (GMST) is estimated to been 27.0−37.1°C during the late Paleocene to early Eocene based on $\delta^{18}O$ planktonic, TEX_{86} and multi-method experiment [21, 22]. The range of clumped isotope temperatures from the Mopola section is nearly identical to the GMST, suggesting that there is a minimal temperature decreases from sea level to the Luolong Basin. This indicates a relatively low elevation and approximates to our oxygen paleoelevation results.

5. Figures S1 to S7

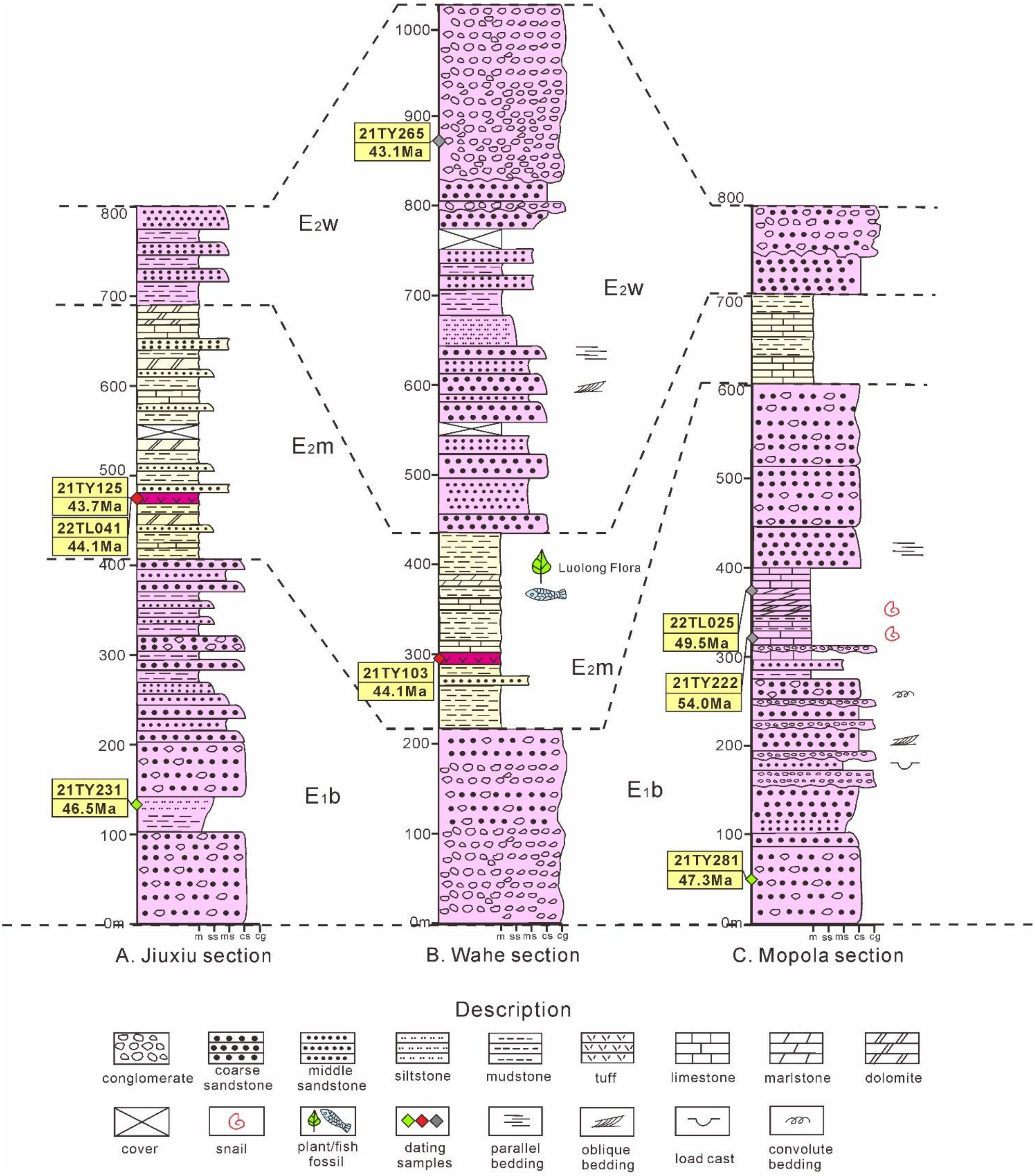

Figure S1. Three stratigraphic columns in the Luolong Basin including the Jiuxiu section, the Wahe section and the Mopola section. The corresponding positions of these three sections can be found in Fig. 1b.

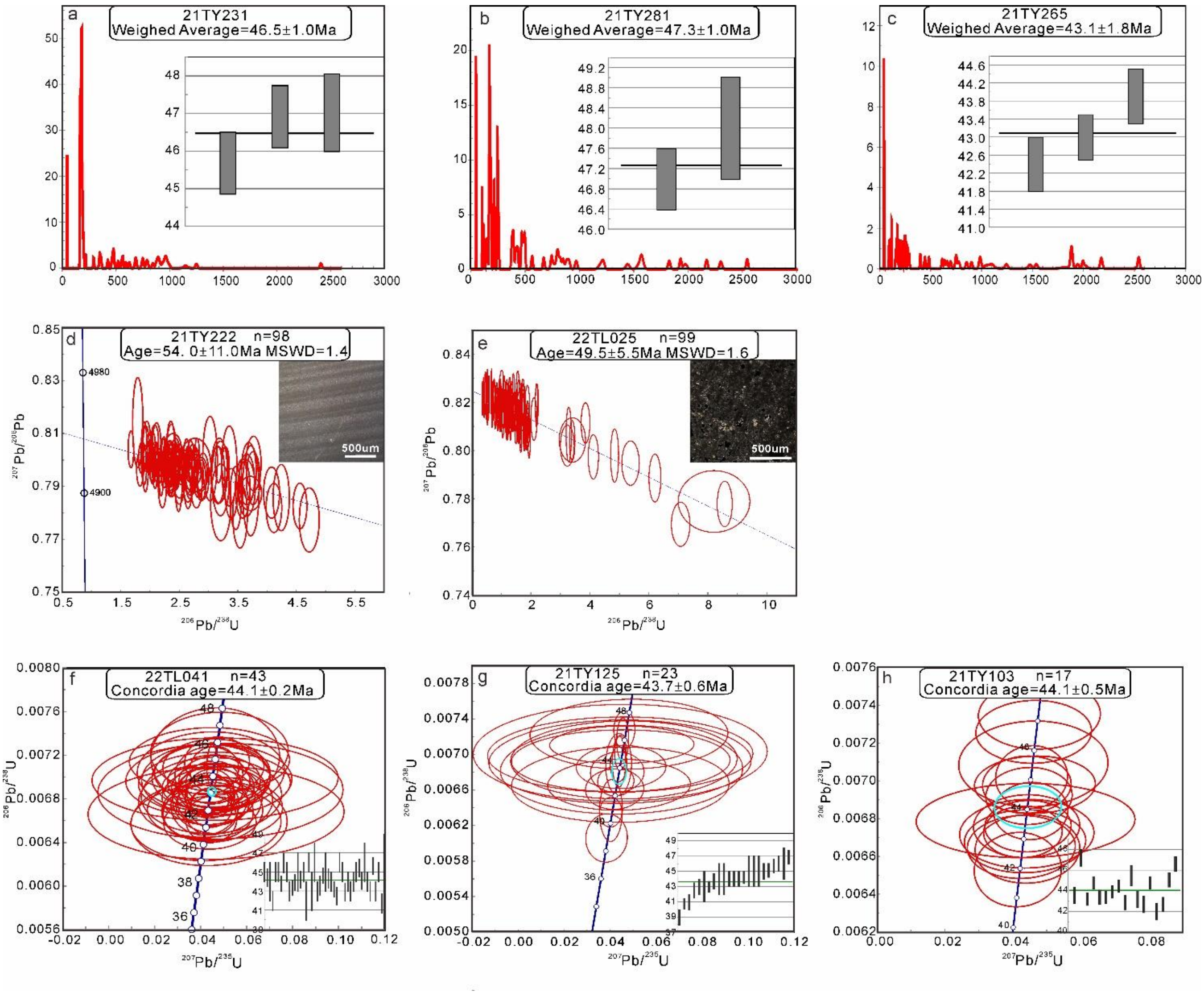

Figure S2. U−Pb dating results of the Luolong Basin. Fig. S2a is the sandstone sample (21TY231) from the lower part of the Buxu Formation, Jiuxiu section. Fig. S2b is the sandstone sample (21TY281) collected at the lower part of the Buxu Formation, Mopola section. Fig. S2c is the sandstone sample (21TY265) at the upper part of the Wahe Formation, Wahe section. Fig. S2d and Fig. S2e are the limestone samples (21TY222 and 22TL025) from the upper part of the Buxu Formation, Mopola section. Inserted pictures show the petrographic characteristics within cross-polarized light. Fig. S2f and Fig. S2g are the tuff samples (22TL041 and 21TY125) at the Meiduo Formation, Jiuxiu section. Fig. S2h is the tuff sample (21TY103) at the Meiduo Formation, Wahe section.

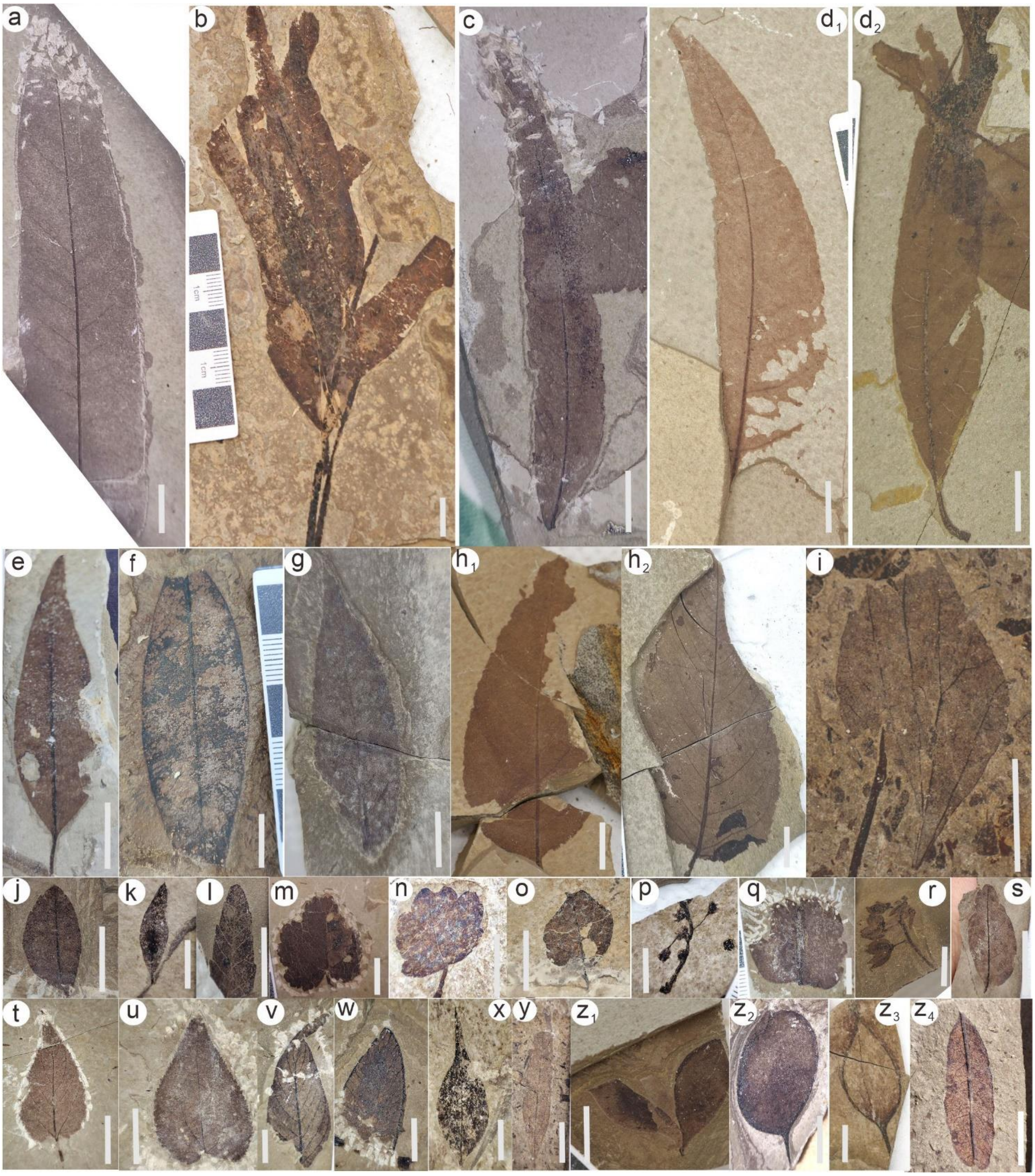

Figure S3. Plant fossils of the middle Eocene Luolong Flora in the Luolong Basin. Scale bars equal to 1cm. (a) OTU5. (b) OTU40. (h_1−h_2) *Zelkova* sp., OTU31. (j) OTU13. (m) OTU50. (n) *Populus* sp., OTU19. (s) OTU44. (t) *Populus* sp., OTU20. (u) OTU17. (v) OTU32. (x) Leguminophyllum (OTU43). (z_1−z_3) *Podocarpium* sp.

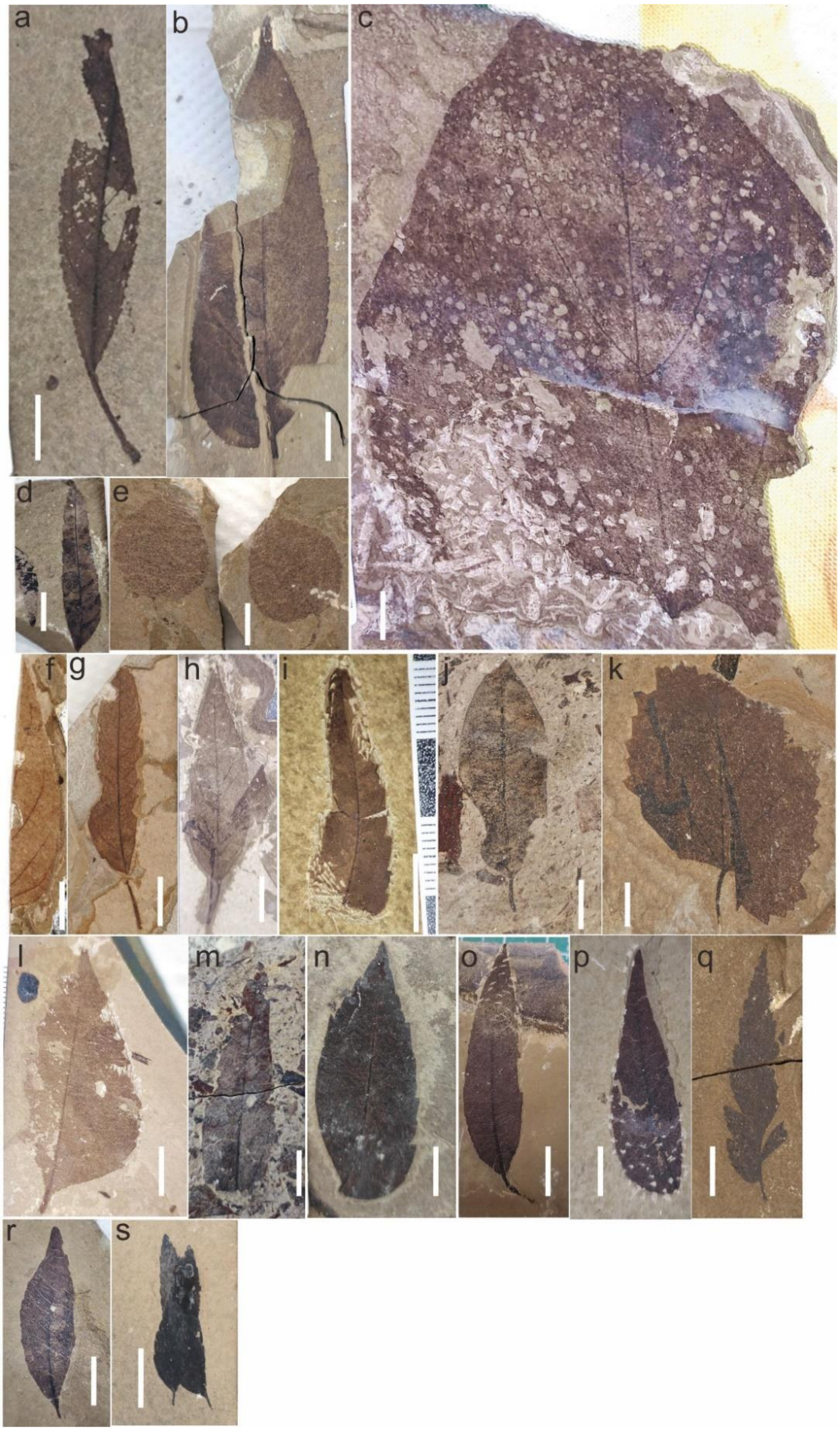

Figure S4. Plant fossils of the middle Eocene Luolong Flora in the Luolong Basin. Scale bars equal to 1cm. (a) OTU29. (b) OTU3. (c) *Cercis* sp., OTU51. (d) *Salix* sp., OTU30. (e) *Exbucklandia* sp., OTU46. (f) OTU49. (g) Anacardiaceae, OTU5. (h) OTU7. (j) OTU14. (k) *Populus* sp., OTU18. (l) OTU26. (m) OTU15. (n) OTU38. (o) OTU37. (p) OTU42. (q) OTU47. (r) OTU23. (s) *Hemiptelea* sp., OTU25.

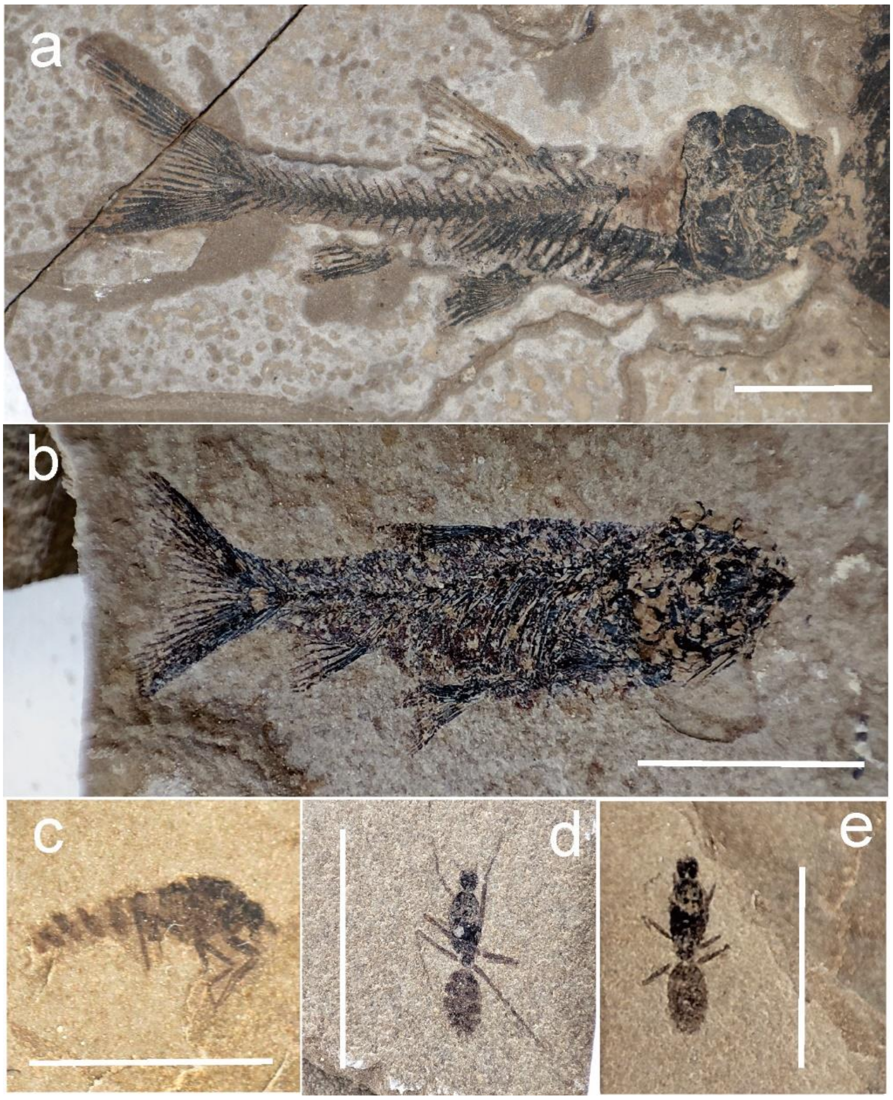

Figure S5. Fish and insect fossils from the same layer of the Luolong Flora in the Luolong Basin. Scale bars equal to 1 cm.

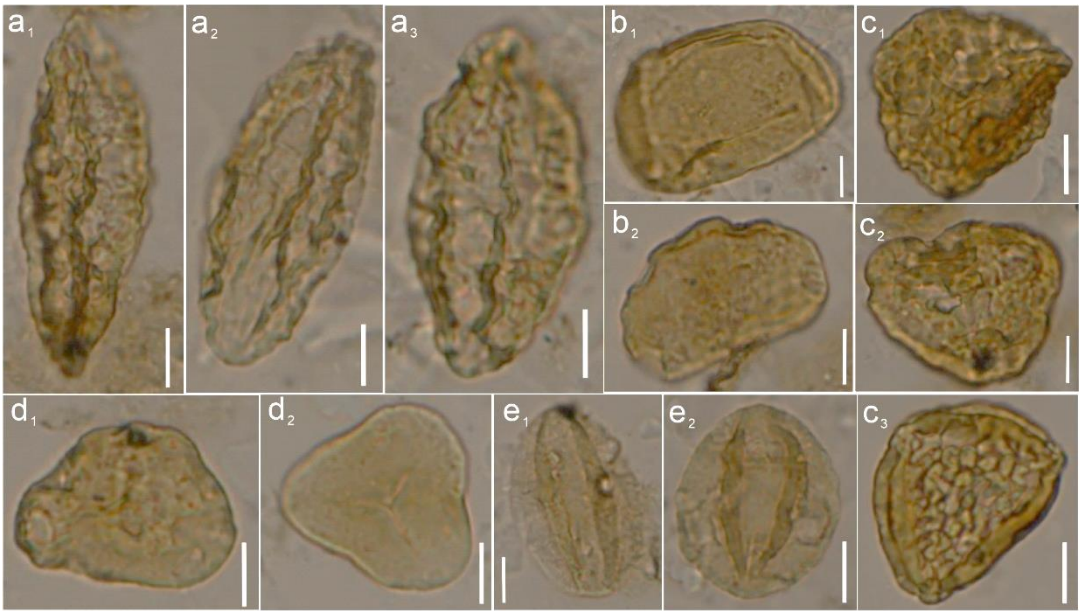

Figure S6. Photographs of pollen grains from the Buxu Formation in the Luolong Basin. Scale bars equal to 20 µm. (a_1–a_3) *Ephedripites*. (b_1–b_2) *Polypodiaceaesporites*. (c_1–c_3) *Pterisisporites*. (d_1–d_2) *Deltoidospora*. (e_1–e_2) *Cornaceoipollenites*.

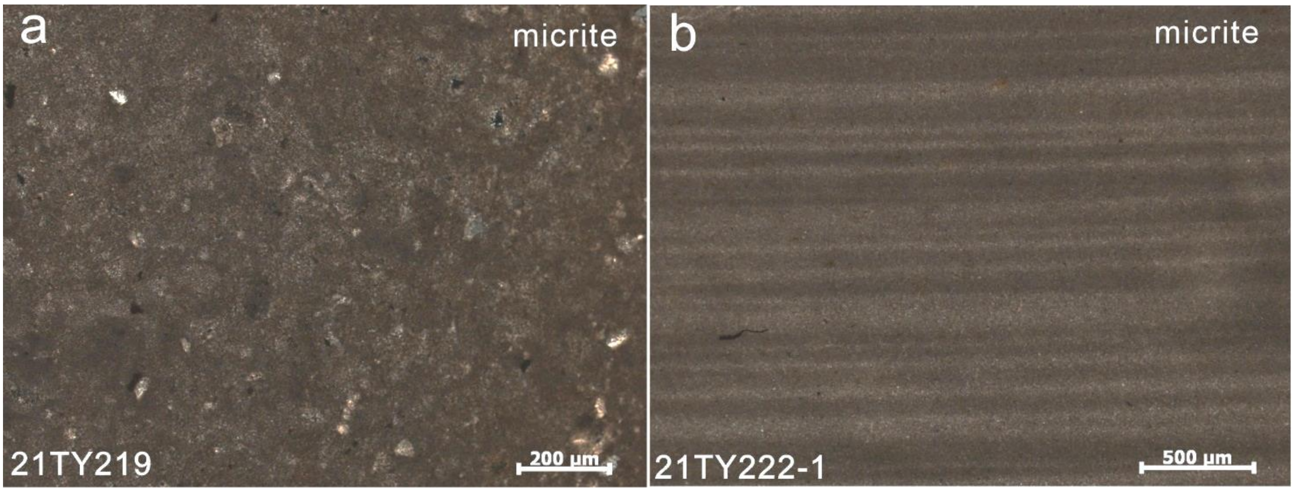

Figure S7. Petrographic images of the limestone samples from the Buxu Formation, Mopola section, Luolong Basin. All photos are under cross-polarized light. (a) Petrographic image of 21TY219 reveals the micrite texture. (b) Petrographic picture of 21TY222-1 displays a micrite texture with parallel bedding.

6. Tables S1 to S8

Table S1 Zircon LA-ICP-MS U–Pb in-situ results

Analysis	corrected ratios								corrected ages							
	$^{207}Pb/^{206}Pb$	1σ	$^{207}Pb/^{235}U$	1σ	$^{206}Pb/^{238}U$	1σ	$^{208}Pb/^{232}Th$	1σ	$^{207}Pb/^{206}Pb$	1σ	$^{207}Pb/^{235}U$	1σ	$^{206}Pb/^{238}U$	1σ	$^{208}Pb/^{232}Th$	1σ
**22TL041**																
22TL041-001	0.04811	0.02281	0.046	0.02178	0.00694	0.00019	0.00241	0.00015	105.0	770.0	46.0	21.0	45.0	1.0	49.0	3.0
22TL041-002	0.04797	0.01557	0.0457	0.01482	0.00691	0.00014	0.00226	0.00006	98.0	512.0	45.0	14.0	44.4	0.9	46.0	1.0
22TL041-003	0.05135	0.03721	0.04864	0.03519	0.00687	0.00029	0.00327	0.00035	257.0	1074.0	48.0	34.0	44.0	2.0	66.0	7.0
22TL041-004	0.04855	0.01466	0.04404	0.01326	0.00658	0.00016	0.00256	0.0001	126.0	471.0	44.0	13.0	42.0	1.0	52.0	2.0
22TL041-005	0.0478	0.01393	0.04582	0.01333	0.00696	0.00016	0.00266	0.00013	89.0	449.0	45.0	13.0	45.0	1.0	54.0	3.0
22TL041-006	0.04503	0.0101	0.04358	0.00974	0.00702	0.00017	0.00234	0.00011	-18.0	322.0	43.0	9.0	45.0	1.0	47.0	2.0
22TL041-007	0.04694	0.00756	0.04411	0.00707	0.00682	0.00013	0.00242	0.00008	46.0	267.0	44.0	7.0	43.8	0.8	49.0	2.0
22TL041-008	0.04792	0.0095	0.04715	0.00931	0.00714	0.00015	0.00245	0.00011	95.0	327.0	47.0	9.0	45.9	1.0	49.0	2.0
22TL041-009	0.04536	0.02487	0.04356	0.02385	0.00697	0.00022	0.00249	0.00023	-1.0	864.0	43.0	23.0	45.0	1.0	50.0	5.0
22TL041-010	0.04716	0.01407	0.04337	0.0129	0.00667	0.00017	0.00253	0.0001	57.0	455.0	43.0	13.0	43.0	1.0	51.0	2.0
22TL041-011	0.04859	0.01134	0.04518	0.01053	0.00675	0.00013	0.00256	0.00007	128.0	383.0	45.0	10.0	43.4	0.8	52.0	1.0
22TL041-012	0.04583	0.007	0.04321	0.00657	0.00684	0.00013	0.00222	0.00007	-11.0	249.0	43.0	6.0	43.9	0.8	45.0	1.0
22TL041-013	0.0481	0.03674	0.0463	0.03533	0.00698	0.00025	0.00284	0.00031	104.0	1102.0	46.0	34.0	45.0	2.0	57.0	6.0
22TL041-014	0.04685	0.00983	0.04444	0.0093	0.00688	0.00014	0.0027	0.00009	42.0	335.0	44.0	9.0	44.2	0.9	54.0	2.0
22TL041-015	0.04402	0.01921	0.04236	0.01846	0.00698	0.00021	0.00235	0.00016	-70.0	652.0	42.0	18.0	45.0	1.0	47.0	3.0
22TL041-016	0.04822	0.02478	0.04369	0.0224	0.00658	0.00026	0.00241	0.00016	110.0	831.0	43.0	22.0	42.0	2.0	49.0	3.0
22TL041-017	0.04446	0.02337	0.04384	0.02302	0.00715	0.0002	0.00294	0.00016	-47.0	814.0	44.0	22.0	46.0	1.0	59.0	3.0
22TL041-018	0.05087	0.04005	0.04673	0.03674	0.00667	0.00028	0.0038	0.00024	235.0	1173.0	46.0	36.0	43.0	2.0	77.0	5.0
22TL041-019	0.04684	0.01873	0.04638	0.01846	0.00718	0.0003	0.00282	0.00024	41.0	592.0	46.0	18.0	46.0	2.0	57.0	5.0
22TL041-020	0.04514	0.01054	0.04191	0.00973	0.00674	0.00018	0.00281	0.00014	-12.0	334.0	42.0	9.0	43.0	1.0	57.0	3.0
22TL041-021	0.04824	0.0108	0.04509	0.01006	0.00678	0.00015	0.00222	0.00011	111.0	361.0	45.0	10.0	43.6	1.0	45.0	2.0
22TL041-022	0.04742	0.00815	0.0454	0.00779	0.00695	0.00013	0.00279	0.00008	70.0	285.0	45.0	8.0	44.6	0.8	56.0	2.0
22TL041-023	0.04574	0.02176	0.04445	0.0211	0.00705	0.00024	0.00231	0.00023	-16.0	739.0	44.0	21.0	45.0	2.0	47.0	5.0
22TL041-024	0.04765	0.01254	0.04533	0.0119	0.0069	0.00015	0.00237	0.00009	82.0	409.0	45.0	12.0	44.3	1.0	48.0	2.0
22TL041-025	0.04509	0.02063	0.04215	0.01924	0.00678	0.00022	0.00245	0.00019	-15.0	697.0	42.0	19.0	44.0	1.0	49.0	4.0
22TL041-026	0.04805	0.00657	0.04452	0.00606	0.00672	0.00012	0.00248	0.00006	102.0	249.0	44.0	6.0	43.2	0.8	50.0	1.0

22TL041-027	0.04901	0.00808	0.04464	0.00732	0.00661	0.00014	0.00266	0.00009	148.0	292.0	44.0	7.0	42.5	0.9	54.0	2.0
22TL041-028	0.04665	0.01491	0.04556	0.01453	0.00709	0.00017	0.00255	0.00011	31.0	486.0	45.0	14.0	46.0	1.0	51.0	2.0
22TL041-029	0.04618	0.01762	0.04458	0.01697	0.007	0.0002	0.00273	0.00015	7.0	576.0	44.0	16.0	45.0	1.0	55.0	3.0
22TL041-030	0.04859	0.00813	0.04476	0.00746	0.00668	0.00014	0.00241	0.00009	128.0	293.0	44.0	7.0	42.9	0.9	49.0	2.0
22TL041-031	0.04753	0.02146	0.0447	0.02013	0.00683	0.00023	0.00251	0.00019	76.0	708.0	44.0	20.0	44.0	1.0	51.0	4.0
22TL041-032	0.047	0.01422	0.0432	0.01303	0.00667	0.00017	0.00244	0.0001	49.0	461.0	43.0	13.0	43.0	1.0	49.0	2.0
22TL041-033	0.05047	0.01375	0.0473	0.01282	0.0068	0.00021	0.00272	0.00019	217.0	435.0	47.0	12.0	44.0	1.0	55.0	4.0
22TL041-034	0.04646	0.01713	0.04508	0.01659	0.00704	0.00018	0.00258	0.00017	22.0	562.0	45.0	16.0	45.0	1.0	52.0	3.0
22TL041-035	0.04718	0.00725	0.04595	0.00702	0.00707	0.00014	0.00262	0.00008	58.0	264.0	46.0	7.0	45.4	0.9	53.0	2.0
22TL041-036	0.04688	0.00723	0.04431	0.0068	0.00686	0.00014	0.0023	0.00006	43.0	259.0	44.0	7.0	44.1	0.9	46.0	1.0
22TL041-037	0.04831	0.0199	0.04814	0.01978	0.00723	0.00024	0.00287	0.00018	114.0	641.0	48.0	19.0	46.0	2.0	58.0	4.0
22TL041-038	0.04622	0.01421	0.04346	0.01333	0.00682	0.00017	0.00254	0.00011	9.0	463.0	43.0	13.0	44.0	1.0	51.0	2.0
22TL041-039	0.05006	0.02104	0.04627	0.01941	0.00671	0.00019	0.00247	0.00013	198.0	681.0	46.0	19.0	43.0	1.0	50.0	3.0
22TL041-040	0.04605	0.0018	0.04507	0.00155	0.0071	0.00013	0.00265	0.00019		82.0	45.0	2.0	45.6	0.9	53.0	4.0
22TL041-041	0.04895	0.01575	0.0451	0.01447	0.00669	0.00019	0.00298	0.0002	145.0	498.0	45.0	14.0	43.0	1.0	60.0	4.0
22TL041-042	0.04793	0.01074	0.04593	0.01025	0.00695	0.00016	0.00247	0.0001	96.0	359.0	46.0	10.0	45.0	1.0	50.0	2.0
22TL041-043	0.04668	0.02385	0.04231	0.02157	0.00658	0.00023	0.00256	0.00017	33.0	818.0	42.0	21.0	42.0	1.0	52.0	3.0
**21TY103**																
21TY103-001	0.04668	0.02269	0.04382	0.02128	0.00679	0.00013	0.00253	0.00024	33	796	44	21	43.6	0.8	51	5
21TY103-002	0.04717	0.0117	0.04789	0.01186	0.00735	0.00012	0.00245	0.00027	58	391	47	11	47.2	0.8	49	5
21TY103-003	0.04631	0.00826	0.04305	0.00766	0.00673	0.00009	0.00248	0.00025	14	293	43	7	43.2	0.6	50	5
21TY103-004	0.04585	0.00896	0.04419	0.00862	0.00698	0.0001	0.00275	0.00028	-10	312	44	8	44.8	0.6	56	6
21TY103-005	0.0472	0.01108	0.0441	0.01033	0.00676	0.00011	0.00253	0.00027	59	378	44	10	43.4	0.7	51	5
21TY103-006	0.04771	0.00902	0.04451	0.0084	0.00676	0.00009	0.00225	0.00025	85	320	44	8	43.4	0.6	45	5
21TY103-007	0.04799	0.00767	0.04535	0.00722	0.00684	0.0001	0.00233	0.00027	99	282	45	7	43.9	0.6	47	5
21TY103-008	0.04694	0.00882	0.04482	0.0084	0.00692	0.0001	0.0025	0.00032	46	309	45	8	44.5	0.6	50	6
21TY103-009	0.04734	0.01282	0.04367	0.01181	0.00668	0.00011	0.00248	0.00029	66	427	43	11	42.9	0.7	50	6
21TY103-010	0.04716	0.01488	0.04611	0.01452	0.00709	0.00015	0.00269	0.00023	57	490	46	14	45.5	1	54	5
21TY103-011	0.04763	0.01412	0.04417	0.01307	0.00672	0.00012	0.00177	0.00014	81	465	44	13	43.2	0.8	36	3
21TY103-012	0.04845	0.01114	0.04436	0.01017	0.00664	0.00012	0.00185	0.00009	121	378	44	10	42.7	0.8	37	2
21TY103-013	0.0484	0.01223	0.04657	0.01175	0.00698	0.0001	0.00197	0.00013	119	416	46	11	44.8	0.6	40	3
21TY103-014	0.04611	0.00945	0.04152	0.00848	0.00653	0.00013	0.0021	0.00014	3	320	41	8	42	0.8	42	3
21TY103-015	0.04639	0.01147	0.04257	0.01051	0.00665	0.00011	0.00208	0.00013	18	384	42	10	42.7	0.7	42	3

21TY103-016	0.0471	0.00727	0.04558	0.007	0.00702	0.00011	0.00211	0.00013	54	264	45	7	45.1	0.7	43	3		
21TY103-017	0.04618	0.01127	0.0462	0.01126	0.00726	0.00011	0.00214	0.00017	7	380	46	11	46.6	0.7	43	3		
**21TY125**																		
21TY125-001	0.04605	0.00727	0.0385	0.00598	0.00606	0.00018	0.01468	0.0005		282.0	38.0	6.0	39.0	1.0	294.0	10.0		
21TY125-002	0.04789	0.03708	0.04504	0.03481	0.00683	0.00033	0.00305	0.00018	94.0	1097.0	45.0	34.0	44.0	2.0	62.0	4.0		
21TY125-003	0.04817	0.03406	0.04608	0.03253	0.00694	0.00029	0.00271	0.00032	108.0	1025.0	46.0	32.0	45.0	2.0	55.0	6.0		
21TY125-004	0.04605	0.00201	0.0455	0.00172	0.00717	0.00016	0.00448	0.00019		93.0	45.0	2.0	46.0	1.0	90.0	4.0		
21TY125-005	0.04605	0.00924	0.04199	0.00837	0.00661	0.00015	0.01369	0.00034		342.0	42.0	8.0	42.5	1.0	275.0	7.0		
21TY125-006	0.04787	0.02241	0.04392	0.02053	0.00666	0.00019	0.00258	0.00019	93.0	754.0	44.0	20.0	43.0	1.0	52.0	4.0		
21TY125-007	0.04845	0.03412	0.04449	0.03127	0.00666	0.00028	0.00373	0.00026	121.0	1022.0	44.0	30.0	43.0	2.0	75.0	5.0		
21TY125-008	0.04605	0.00378	0.04028	0.00323	0.00635	0.00011	0.00823	0.00026		180.0	40.0	3.0	40.8	0.7	166.0	5.0		
21TY125-009	0.04655	0.0396	0.04435	0.03768	0.00692	0.00031	0.00318	0.0003	26.0	1100.0	44.0	37.0	44.0	2.0	64.0	6.0		
21TY125-010	0.04605	0.00196	0.04457	0.0017	0.00702	0.00013	0.0051	0.00024		91.0	44.0	2.0	45.1	0.9	103.0	5.0		
21TY125-011	0.04605	0.00314	0.04624	0.00303	0.00728	0.00014	0.00664	0.00021		150.0	46.0	3.0	46.8	0.9	134.0	4.0		
21TY125-012	0.04845	0.02858	0.04826	0.02842	0.00723	0.00026	0.00286	0.00016	121.0	960.0	48.0	28.0	46.0	2.0	58.0	3.0		
21TY125-013	0.04605	0.00237	0.04391	0.0019	0.00692	0.00019	0.00546	0.00043		111.0	44.0	2.0	44.0	1.0	110.0	9.0		
21TY125-014	0.04605	0.03952	0.04446	0.03812	0.007	0.00028	0.06236	0.0014		1134.0	44.0	37.0	45.0	2.0	1223.0	27.0		
21TY125-015	0.04605	0.0083	0.04317	0.00772	0.0068	0.00015	0.00384	0.00025		311.0	43.0	8.0	43.7	1.0	77.0	5.0		
21TY125-016	0.04605	0.00418	0.04375	0.0037	0.00689	0.00023	0.00906	0.00058		198.0	43.0	4.0	44.0	1.0	182.0	12.0		
21TY125-017	0.04605	0.02118	0.04515	0.02075	0.00711	0.00014	0.02569	0.0006		773.0	45.0	20.0	45.7	0.9	513.0	12.0		
21TY125-018	0.04596	0.04371	0.04447	0.04224	0.00702	0.00036	0.00288	0.0003	-4.0	1151.0	44.0	41.0	45.0	2.0	58.0	6.0		
21TY125-019	0.04682	0.03293	0.0447	0.03141	0.00693	0.00023	0.00259	0.0002	40.0	1017.0	44.0	31.0	45.0	1.0	52.0	4.0		
21TY125-020	0.04605	0.00266	0.04371	0.00238	0.00689	0.00013	0.00587	0.00026		126.0	43.0	2.0	44.2	0.8	118.0	5.0		
21TY125-021	0.0474	0.01365	0.04484	0.01288	0.00686	0.00016	0.00221	0.00012	69.0	440.0	45.0	13.0	44.0	1.0	45.0	2.0		
21TY125-022	0.04605	0.0057	0.04253	0.00522	0.0067	0.00011	0.0114	0.00035		238.0	42.0	5.0	43.0	0.7	229.0	7.0		
21TY125-023	0.04605	0.00411	0.04043	0.00331	0.00637	0.00023	0.00764	0.00044		195.0	40.0	3.0	41.0	1.0	154.0	9.0		
Analysis	corrected ratios								corrected ages								used age	1σ
	$^{207}Pb/^{206}Pb$	1σ	$^{207}Pb/^{235}U$	1σ	$^{206}Pb/^{238}U$	1σ	$^{208}Pb/^{232}Th$	1σ	$^{207}Pb/^{206}Pb$	1σ	$^{207}Pb/^{235}U$	1σ	$^{206}Pb/^{238}U$	1σ	$^{208}Pb/^{232}Th$	1σ		
**21TY231**																		
2021TY231-001	0.04679	0.0078	0.04729	0.0078	0.00739	0.00019	0.00252	0.00015	39.0	264.0	47.0	8.0	47.0	1.0	51.0	3.0	47	1
2021TY231-002	0.06186	0.001	1.04741	0.01726	0.12383	0.00147	0.03924	0.00037	669.0	17.0	728.0	9.0	753.0	8.0	778.0	7.0	753	8

2021TY231-003	0.0513	0.0011	0.32752	0.00707	0.04668	0.00057	0.02591	0.00045	254.0	28.0	288.0	5.0	294.0	4.0	517.0	9.0	294	4
2021TY231-004	0.06107	0.00117	0.8038	0.01567	0.09622	0.00114	0.03355	0.00031	642.0	23.0	599.0	9.0	592.0	7.0	667.0	6.0	592	7
2021TY231-005	0.06165	0.00174	0.94598	0.02673	0.11218	0.0014	0.04819	0.00068	662.0	39.0	676.0	14.0	685.0	8.0	951.0	13.0	685	8
2021TY231-006	0.05046	0.00419	0.19131	0.01574	0.02771	0.00047	0.00801	0.00019	216.0	154.0	178.0	13.0	176.0	3.0	161.0	4.0	176	3
2021TY231-007	0.05122	0.00153	0.21348	0.00634	0.03046	0.00039	0.00773	0.00019	251.0	45.0	196.0	5.0	193.0	2.0	156.0	4.0	193	2
2021TY231-008	0.05183	0.00285	0.20333	0.01106	0.02865	0.00043	0.00792	0.00016	278.0	97.0	188.0	9.0	182.0	3.0	159.0	3.0	182	3
2021TY231-009	0.04809	0.00404	0.19157	0.01602	0.02909	0.00043	0.00685	0.00018	104.0	158.0	178.0	14.0	185.0	3.0	138.0	4.0	185	3
2021TY231-010	0.05432	0.00335	0.21308	0.01306	0.02864	0.0004	0.00603	0.00009	384.0	113.0	196.0	11.0	182.0	3.0	122.0	2.0	182	3
2021TY231-011	0.08218	0.00089	2.43447	0.02832	0.21626	0.00247	0.05436	0.00045	1250.0	10.0	1253.0	8.0	1262.0	13.0	1070.0	9.0	1250	10
2021TY231-012	0.0471	0.00298	0.18353	0.01149	0.02845	0.00043	0.00831	0.00014	54.0	108.0	171.0	10.0	181.0	3.0	167.0	3.0	181	3
2021TY231-013	0.05808	0.0015	0.61246	0.01577	0.07698	0.00097	0.02303	0.00023	533.0	35.0	485.0	10.0	478.0	6.0	460.0	5.0	478	6
2021TY231-014	0.04912	0.00246	0.18115	0.00899	0.02691	0.00038	0.00772	0.00026	154.0	88.0	169.0	8.0	171.0	2.0	155.0	5.0	171	2
2021TY231-015	0.05121	0.00257	0.19153	0.00955	0.02729	0.00036	0.00725	0.00015	250.0	91.0	178.0	8.0	174.0	2.0	146.0	3.0	174	2
2021TY231-016	0.05012	0.00226	0.20142	0.00904	0.02931	0.00039	0.00894	0.00016	201.0	79.0	186.0	8.0	186.0	2.0	180.0	3.0	186	2
2021TY231-017	0.0547	0.00516	0.22952	0.02156	0.03061	0.00046	0.00764	0.00022	400.0	185.0	210.0	18.0	194.0	3.0	154.0	4.0	194	3
2021TY231-018	0.0622	0.00118	0.78016	0.01493	0.09148	0.00108	0.01992	0.0008	681.0	22.0	586.0	9.0	564.0	6.0	399.0	16.0	564	6
2021TY231-019	0.04887	0.0036	0.19038	0.01384	0.02841	0.00049	0.00879	0.00019	142.0	131.0	177.0	12.0	181.0	3.0	177.0	4.0	181	3
2021TY231-020	0.05089	0.00248	0.19643	0.00948	0.02814	0.00039	0.00786	0.00011	236.0	86.0	182.0	8.0	179.0	2.0	158.0	2.0	179	2
2021TY231-021	0.07043	0.00089	1.43804	0.01902	0.14884	0.0017	0.04453	0.00051	941.0	12.0	905.0	8.0	894.0	10.0	881.0	10.0	894	10
2021TY231-022	0.07344	0.0015	1.30408	0.02681	0.12942	0.00155	0.02602	0.00034	1026.0	23.0	848.0	12.0	785.0	9.0	519.0	7.0	785	9
2021TY231-023	0.06332	0.00185	0.78708	0.02296	0.09059	0.00112	0.02466	0.00038	719.0	41.0	590.0	13.0	559.0	7.0	492.0	7.0	559	7
2021TY231-024	0.07047	0.00118	1.08476	0.01852	0.11217	0.0013	0.03271	0.00047	942.0	17.0	746.0	9.0	685.0	8.0	651.0	9.0	685	8
2021TY231-025	0.05156	0.00163	0.20128	0.00635	0.02845	0.00034	0.01123	0.00017	266.0	50.0	186.0	5.0	181.0	2.0	226.0	3.0	181	2
2021TY231-026	0.0464	0.00555	0.0453	0.00538	0.00711	0.00013	0.00211	0.00026	18.0	215.0	45.0	5.0	45.7	0.8	43.0	5.0	46	1
2021TY231-027	0.05459	0.00234	0.22296	0.0095	0.02976	0.00038	0.00571	0.00011	395.0	73.0	204.0	8.0	189.0	2.0	115.0	2.0	189	2
2021TY231-028	0.06795	0.00149	1.38351	0.03023	0.14834	0.00181	0.03377	0.00078	867.0	26.0	882.0	13.0	892.0	10.0	671.0	15.0	892	10
2021TY231-029	0.04898	0.00382	0.19496	0.01512	0.02899	0.00043	0.00832	0.00017	147.0	147.0	181.0	13.0	184.0	3.0	167.0	3.0	184	3

2021TY231-030	0.04814	0.00259	0.18051	0.00964	0.0273	0.00036	0.00772	0.00018	106.0	95.0	168.0	8.0	174.0	2.0	155.0	4.0	174	2
2021TY231-031	0.05133	0.00315	0.20105	0.01229	0.02852	0.00039	0.00515	0.00012	256.0	116.0	186.0	10.0	181.0	2.0	104.0	2.0	181	2
2021TY231-032	0.15574	0.00164	9.63945	0.10838	0.45061	0.00507	0.06957	0.00053	2410.0	9.0	2401.0	10.0	2398.0	23.0	1359.0	10.0	2410	9
2021TY231-033	0.06097	0.00107	0.70822	0.01264	0.08456	0.00097	0.03667	0.00043	638.0	20.0	544.0	8.0	523.0	6.0	728.0	8.0	523	6
2021TY231-034	0.04932	0.00195	0.20063	0.00788	0.02961	0.00038	0.00871	0.00013	163.0	68.0	186.0	7.0	188.0	2.0	175.0	3.0	188	2
2021TY231-035	0.04847	0.00161	0.19089	0.00633	0.02866	0.00035	0.00779	0.00007	122.0	55.0	177.0	5.0	182.0	2.0	157.0	1.0	182	2
2021TY231-036	0.05825	0.00134	0.46319	0.01058	0.05786	0.00069	0.06802	0.0008	539.0	29.0	386.0	7.0	363.0	4.0	1330.0	15.0	363	4
2021TY231-037	0.0707	0.00078	1.64927	0.01918	0.16972	0.00188	0.07958	0.0012	949.0	11.0	989.0	7.0	1011.0	10.0	1548.0	22.0	949	11
2021TY231-038	0.05055	0.00999	0.18797	0.03679	0.02705	0.00081	0.03965	0.01511	220.0	330.0	175.0	31.0	172.0	5.0	786.0	294.0	172	5
2021TY231-039	0.05101	0.00348	0.22301	0.01513	0.0318	0.00043	0.01009	0.00019	241.0	130.0	204.0	13.0	202.0	3.0	203.0	4.0	202	3
2021TY231-040	0.05022	0.00152	0.18929	0.00573	0.02741	0.00032	0.00806	0.00009	205.0	48.0	176.0	5.0	174.0	2.0	162.0	2.0	174	2
2021TY231-041	0.06625	0.00149	1.27672	0.02882	0.14012	0.00165	0.0393	0.0005	814.0	28.0	835.0	13.0	845.0	9.0	779.0	10.0	845	9
2021TY231-042	0.0602	0.00156	0.61806	0.01597	0.07465	0.00088	0.02352	0.00041	611.0	36.0	489.0	10.0	464.0	5.0	470.0	8.0	464	5
2021TY231-043	0.04986	0.00177	0.19992	0.00702	0.02915	0.00037	0.00681	0.00018	188.0	58.0	185.0	6.0	185.0	2.0	137.0	4.0	185	2
2021TY231-044	0.05068	0.00136	0.18537	0.00493	0.02659	0.00032	0.00586	0.00006	226.0	39.0	173.0	4.0	169.0	2.0	118.0	1.0	169	2
2021TY231-045	0.06626	0.00101	1.37329	0.02122	0.15066	0.00171	0.04183	0.0004	815.0	15.0	878.0	9.0	905.0	10.0	828.0	8.0	905	10
2021TY231-046	0.05135	0.00334	0.19827	0.01264	0.02806	0.00049	0.00983	0.00038	257.0	114.0	184.0	11.0	178.0	3.0	198.0	8.0	178	3
2021TY231-047	0.06242	0.00126	1.05592	0.02128	0.12296	0.00144	0.03646	0.00028	689.0	24.0	732.0	11.0	748.0	8.0	724.0	5.0	748	8
2021TY231-048	0.05353	0.0023	0.20479	0.00867	0.0278	0.00039	0.00538	0.00015	351.0	70.0	189.0	7.0	177.0	2.0	108.0	3.0	177	2
2021TY231-049	0.05092	0.00337	0.19433	0.0127	0.02773	0.00044	0.00531	0.00015	237.0	120.0	180.0	11.0	176.0	3.0	107.0	3.0	176	3
2021TY231-050	0.0715	0.00108	1.60616	0.02465	0.16323	0.00184	0.04953	0.00045	972.0	15.0	973.0	10.0	975.0	10.0	977.0	9.0	975	10
2021TY231-051	0.07095	0.00106	1.57199	0.02377	0.16098	0.00181	0.04419	0.00071	956.0	15.0	959.0	9.0	962.0	10.0	874.0	14.0	962	10
2021TY231-052	0.0696	0.00099	1.39347	0.02026	0.14547	0.00163	0.04113	0.00036	917.0	14.0	886.0	9.0	876.0	9.0	815.0	7.0	876	9
2021TY231-053	0.05458	0.00419	0.20297	0.01547	0.02702	0.0004	0.00581	0.00016	395.0	146.0	188.0	13.0	172.0	3.0	117.0	3.0	172	3
2021TY231-054	0.05177	0.00187	0.19998	0.00717	0.02806	0.00034	0.02669	0.00088	275.0	60.0	185.0	6.0	178.0	2.0	532.0	17.0	178	2
2021TY231-055	0.05207	0.00162	0.22381	0.00689	0.03121	0.00039	0.00603	0.00015	288.0	47.0	205.0	6.0	198.0	2.0	122.0	3.0	198	2
2021TY231-056	0.05613	0.00187	0.52724	0.01736	0.06822	0.00085	0.01723	0.00025	458.0	51.0	430.0	12.0	425.0	5.0	345.0	5.0	425	5

2021TY231-057	0.04965	0.00102	0.17883	0.00366	0.02615	0.0003	0.01597	0.00088	179.0	27.0	167.0	3.0	166.0	2.0	320.0	18.0	166	2
2021TY231-058	0.04597	0.00275	0.16815	0.00989	0.02656	0.00043	0.00836	0.00025	-4.0	96.0	158.0	9.0	169.0	3.0	168.0	5.0	169	3
2021TY231-059	0.04992	0.0015	0.19486	0.00577	0.02834	0.00035	0.01647	0.00091	191.0	46.0	181.0	5.0	180.0	2.0	330.0	18.0	180	2
2021TY231-060	0.0781	0.0014	2.08048	0.03751	0.19313	0.00227	0.06951	0.00648	1149.0	18.0	1142.0	12.0	1138.0	12.0	1358.0	122.0	1149	18
2021TY231-061	0.04915	0.00216	0.19166	0.00838	0.02827	0.00036	0.00925	0.00089	155.0	78.0	178.0	7.0	180.0	2.0	186.0	18.0	180	2
2021TY231-062	0.05063	0.0026	0.18286	0.00928	0.02619	0.00037	0.00971	0.00094	224.0	91.0	171.0	8.0	167.0	2.0	195.0	19.0	167	2
2021TY231-063	0.05053	0.00229	0.19123	0.00864	0.02745	0.00034	0.01025	0.00097	219.0	82.0	178.0	7.0	175.0	2.0	206.0	19.0	175	2
2021TY231-064	0.05236	0.0023	0.19067	0.00834	0.02641	0.00032	0.00837	0.00083	301.0	78.0	177.0	7.0	168.0	2.0	168.0	17.0	168	2
2021TY231-065	0.05406	0.00323	0.4123	0.02448	0.05532	0.00075	0.01769	0.00171	374.0	110.0	351.0	18.0	347.0	5.0	354.0	34.0	347	5
2021TY231-066	0.04605	0.00291	0.21913	0.01355	0.03451	0.00046	0.01281	0.00164		139.0	201.0	11.0	219.0	3.0	257.0	33.0	219	3
2021TY231-067	0.0642	0.00115	0.90291	0.01647	0.10205	0.00116	0.04647	0.00455	748.0	20.0	653.0	9.0	626.0	7.0	918.0	88.0	626	7
2021TY231-068	0.06941	0.00187	1.48864	0.03996	0.15563	0.00195	0.04822	0.00479	911.0	35.0	926.0	16.0	932.0	11.0	952.0	92.0	932	11
2021TY231-069	0.04801	0.00382	0.17015	0.01345	0.02573	0.00038	0.00948	0.00101	100.0	148.0	160.0	12.0	164.0	2.0	191.0	20.0	164	2
2021TY231-070	0.05277	0.00233	0.19401	0.00844	0.02669	0.00037	0.00983	0.00116	319.0	74.0	180.0	7.0	170.0	2.0	198.0	23.0	170	2
2021TY231-071	0.06919	0.00084	1.53674	0.01967	0.16127	0.0018	0.05506	0.00587	904.0	12.0	945.0	8.0	964.0	10.0	1083.0	112.0	964	10
2021TY231-072	0.07391	0.00151	1.33658	0.0275	0.13133	0.00152	0.05367	0.00581	1039.0	23.0	862.0	12.0	795.0	9.0	1057.0	111.0	795	9
2021TY231-073	0.04682	0.00437	0.17876	0.01656	0.02773	0.00045	0.01068	0.00121	40.0	175.0	167.0	14.0	176.0	3.0	215.0	24.0	176	3
2021TY231-074	0.05183	0.00388	0.18994	0.01417	0.02662	0.00037	0.00897	0.00103	278.0	145.0	177.0	12.0	169.0	2.0	180.0	21.0	169	2
2021TY231-075	0.05335	0.00336	0.41365	0.02559	0.05624	0.00067	0.01761	0.00021	344.0	146.0	352.0	18.0	353.0	4.0	353.0	4.0	353	4
2021TY231-076	0.05103	0.00456	0.20215	0.01778	0.02873	0.00047	0.00905	0.00032	242.0	204.0	187.0	15.0	183.0	3.0	182.0	6.0	183	3
2021TY231-077	0.04605	0.00998	0.04635	0.01001	0.0073	0.00013	0.00242	0.0007		359.0	46.0	10.0	46.9	0.8	49.0	14.0	47	1
2021TY231-078	0.05731	0.00723	0.6056	0.07596	0.07682	0.00141	0.03431	0.00445	503.0	248.0	481.0	48.0	477.0	8.0	682.0	87.0	477	8
2021TY231-079	0.07555	0.00122	1.73279	0.02865	0.16676	0.00193	0.05936	0.00762	1083.0	16.0	1021.0	11.0	994.0	11.0	1166.0	145.0	994	11
2021TY231-080	0.05211	0.00326	0.18579	0.01137	0.02586	0.00034	0.00812	0.00014	290.0	145.0	173.0	10.0	165.0	2.0	163.0	3.0	165	2
2021TY231-081	0.05453	0.0039	0.19858	0.0141	0.02648	0.00038	0.00924	0.00123	393.0	135.0	184.0	12.0	168.0	2.0	186.0	25.0	168	2
2021TY231-082	0.05079	0.0047	0.18735	0.01722	0.02683	0.00043	0.00851	0.00116	231.0	178.0	174.0	15.0	171.0	3.0	171.0	23.0	171	3
2021TY231-083	0.05631	0.00339	0.59573	0.03566	0.07696	0.00108	0.02584	0.00355	465.0	108.0	475.0	23.0	478.0	6.0	516.0	70.0	478	6

2021TY231-084	0.05239	0.00343	0.20408	0.01318	0.02834	0.00045	0.00868	0.00122	302.0	119.0	189.0	11.0	180.0	3.0	175.0	24.0	180	3
**21TY265**																		
21TY265-001	0.08194	0.00125	2.41199	0.03638	0.21348	0.00227	0.04612	0.00142	1244.0	14.0	1246.0	11.0	1247.0	12.0	911.0	27.0	1244	14
21TY265-002	0.05188	0.00775	0.11665	0.01733	0.01631	0.0003	0.00359	0.00018	280.0	292.0	112.0	16.0	104.0	2.0	72.0	4.0	104	2
21TY265-003	0.06674	0.0047	0.99668	0.06983	0.10831	0.00137	0.02726	0.00074	830.0	126.0	702.0	36.0	663.0	8.0	544.0	15.0	663	8
21TY265-004	0.06928	0.00193	1.22553	0.03345	0.12829	0.00152	0.03741	0.00117	907.0	37.0	812.0	15.0	778.0	9.0	742.0	23.0	778	9
21TY265-005	0.07425	0.00157	1.85376	0.03841	0.18106	0.00202	0.04622	0.00129	1048.0	24.0	1065.0	14.0	1073.0	11.0	913.0	25.0	1048	24
21TY265-006	0.11512	0.00146	4.91735	0.0616	0.30979	0.00322	0.0621	0.00167	1882.0	10.0	1805.0	11.0	1740.0	16.0	1218.0	32.0	1882	10
21TY265-007	0.04472	0.0031	0.04216	0.0029	0.00684	0.00009	0.0018	0.00007	-34.0	124.0	42.0	3.0	43.9	0.6	36.0	1.0	44	1
21TY265-008	0.07621	0.00138	1.97414	0.03519	0.18787	0.00205	0.04648	0.00127	1101.0	19.0	1107.0	12.0	1110.0	11.0	918.0	25.0	1101	19
21TY265-009	0.13626	0.00176	6.7741	0.08657	0.36058	0.0038	0.07696	0.00227	2180.0	10.0	2082.0	11.0	1985.0	18.0	1499.0	43.0	2180	10
21TY265-010	0.05393	0.00285	0.25842	0.01355	0.03476	0.00043	0.00915	0.00028	368.0	96.0	233.0	11.0	220.0	3.0	184.0	6.0	220	3
21TY265-011	0.08285	0.00154	1.89682	0.03468	0.16606	0.00182	0.0343	0.00097	1266.0	19.0	1080.0	12.0	990.0	10.0	682.0	19.0	990	10
21TY265-012	0.16845	0.00268	10.48963	0.16351	0.45168	0.00518	0.07753	0.00237	2542.0	12.0	2479.0	14.0	2403.0	23.0	1509.0	44.0	2542	12
21TY265-013	0.11515	0.00133	5.25009	0.0603	0.33072	0.00339	0.06903	0.00202	1882.0	9.0	1861.0	10.0	1842.0	16.0	1349.0	38.0	1882	9
21TY265-014	0.06732	0.00105	0.92672	0.01425	0.09985	0.00105	0.02275	0.00068	848.0	16.0	666.0	8.0	614.0	6.0	455.0	13.0	614	6
21TY265-015	0.04975	0.00211	0.12821	0.00539	0.01869	0.00022	0.00524	0.00016	183.0	76.0	122.0	5.0	119.0	1.0	106.0	3.0	119	1
21TY265-016	0.05243	0.00141	0.31976	0.00847	0.04424	0.0005	0.0119	0.00037	304.0	40.0	282.0	7.0	279.0	3.0	239.0	7.0	279	3
21TY265-017	0.06579	0.00265	1.11617	0.04448	0.12307	0.00145	0.0353	0.00118	800.0	64.0	761.0	21.0	748.0	8.0	701.0	23.0	748	8
21TY265-018	0.09544	0.00142	3.48864	0.05102	0.26517	0.00282	0.0629	0.00201	1537.0	13.0	1525.0	12.0	1516.0	14.0	1233.0	38.0	1537	13
21TY265-019	0.05713	0.00171	0.56193	0.01651	0.07136	0.00084	0.02093	0.00079	497.0	44.0	453.0	11.0	444.0	5.0	419.0	16.0	444	5
21TY265-020	0.05501	0.00271	0.20588	0.01004	0.02715	0.00033	0.00739	0.00025	413.0	87.0	190.0	8.0	173.0	2.0	149.0	5.0	173	2
21TY265-021	0.04791	0.00462	0.04358	0.00417	0.0066	0.0001	0.00252	0.00011	95.0	185.0	43.0	4.0	42.4	0.6	51.0	2.0	42	1
21TY265-022	0.0625	0.00202	0.67135	0.02139	0.07793	0.00091	0.01893	0.00068	691.0	48.0	522.0	13.0	484.0	5.0	379.0	13.0	484	5
21TY265-023	0.05	0.00486	0.17078	0.01637	0.02478	0.00049	0.00808	0.0004	195.0	179.0	160.0	14.0	158.0	3.0	163.0	8.0	158	3
21TY265-024	0.07505	0.00118	1.55371	0.02406	0.1502	0.00158	0.04217	0.00151	1070.0	16.0	952.0	10.0	902.0	9.0	835.0	29.0	902	9
21TY265-025	0.04772	0.00329	0.04786	0.00327	0.00728	0.0001	0.00259	0.00012	85.0	125.0	47.0	3.0	46.8	0.6	52.0	2.0	47	1
21TY265-026	0.07559	0.00161	1.7948	0.03349	0.17222	0.00175	0.05181	0.00052	1084.0	44.0	1044.0	12.0	1024.0	10.0	1021.0	10.0	1084	44
21TY265-027	0.08301	0.00112	1.88421	0.02517	0.16469	0.00171	0.05252	0.00196	1269.0	12.0	1076.0	9.0	983.0	9.0	1035.0	38.0	983	9
21TY265-028	0.0506	0.00383	0.04673	0.00352	0.0067	0.00008	0.0019	0.00008	223.0	149.0	46.0	3.0	43.0	0.5	38.0	2.0	43	1
21TY265-029	0.06692	0.00171	1.1451	0.02899	0.12416	0.00136	0.03762	0.00155	835.0	34.0	775.0	14.0	754.0	8.0	746.0	30.0	754	8
21TY265-030	0.11174	0.00258	5.05571	0.10424	0.32816	0.00343	0.09474	0.00097	1828.0	43.0	1829.0	17.0	1829.0	17.0	1830.0	18.0	1828	43
21TY265-031	0.11919	0.00192	5.47784	0.08672	0.33351	0.00367	0.09759	0.00409	1944.0	14.0	1897.0	14.0	1855.0	18.0	1882.0	75.0	1944	14

21TY265-032	0.16855	0.00198	10.21716	0.11943	0.43988	0.00456	0.12226	0.0051	2543.0	9.0	2455.0	11.0	2350.0	20.0	2331.0	92.0	2543	9
21TY265-033	0.05004	0.00189	0.20069	0.00748	0.0291	0.00035	0.00955	0.00042	197.0	64.0	186.0	6.0	185.0	2.0	192.0	8.0	185	2
21TY265-034	0.04911	0.00262	0.26513	0.01392	0.03918	0.00055	0.01355	0.00067	153.0	94.0	239.0	11.0	248.0	3.0	272.0	13.0	248	3
21TY265-035	0.07077	0.0015	1.38224	0.02871	0.14173	0.00158	0.04592	0.00206	951.0	25.0	881.0	12.0	854.0	9.0	907.0	40.0	854	9
21TY265-036	0.11551	0.00152	5.27949	0.0688	0.3317	0.00348	0.10062	0.00464	1888.0	11.0	1866.0	11.0	1847.0	17.0	1938.0	85.0	1888	11
21TY265-037	0.11504	0.00161	5.46618	0.07539	0.34482	0.00366	0.10397	0.00485	1881.0	12.0	1895.0	12.0	1910.0	18.0	1999.0	89.0	1881	12
21TY265-038	0.13557	0.00181	7.1794	0.09453	0.38432	0.00407	0.13269	0.00626	2171.0	10.0	2134.0	12.0	2096.0	19.0	2518.0	112.0	2171	10
21TY265-039	0.05278	0.00152	0.2303	0.00653	0.03167	0.00036	0.01024	0.00052	319.0	44.0	210.0	5.0	201.0	2.0	206.0	10.0	201	2
21TY265-040	0.12266	0.00147	5.54844	0.0662	0.32829	0.00338	0.12273	0.00594	1995.0	9.0	1908.0	10.0	1830.0	16.0	2340.0	107.0	1995	9
21TY265-041	0.05461	0.00269	0.20837	0.01006	0.02769	0.00039	0.01174	0.00066	396.0	83.0	192.0	8.0	176.0	2.0	236.0	13.0	176	2
21TY265-042	0.055	0.00182	0.30105	0.00977	0.03973	0.00047	0.01632	0.00088	412.0	51.0	267.0	8.0	251.0	3.0	327.0	18.0	251	3
21TY265-043	0.07154	0.00223	1.1246	0.03458	0.1141	0.00131	0.03836	0.00205	973.0	44.0	765.0	17.0	697.0	8.0	761.0	40.0	697	8
21TY265-044	0.05572	0.00135	0.27948	0.00667	0.03641	0.00039	0.01428	0.0008	441.0	34.0	250.0	5.0	231.0	2.0	287.0	16.0	231	2
21TY265-045	0.04605	0.00456	0.16813	0.01653	0.02648	0.00033	0.00916	0.00047		207.0	158.0	14.0	168.0	2.0	184.0	10.0	168	2
21TY265-046	0.04681	0.00346	0.04463	0.00327	0.00692	0.0001	0.00217	0.00014	40.0	133.0	44.0	3.0	44.5	0.6	44.0	3.0	45	1
21TY265-047	0.06089	0.00167	0.87419	0.02353	0.10421	0.00119	0.02993	0.00971	635.0	38.0	638.0	13.0	639.0	7.0	596.0	191.0	639	7
21TY265-048	0.12421	0.0026	6.90615	0.14127	0.40357	0.00487	0.15508	0.0091	2018.0	20.0	2099.0	18.0	2185.0	22.0	2914.0	159.0	2018	20
21TY265-049	0.09236	0.00145	3.43689	0.05329	0.2701	0.00288	0.12089	0.00709	1475.0	15.0	1513.0	12.0	1541.0	15.0	2307.0	128.0	1475	15
21TY265-050	0.0486	0.00907	0.09744	0.01812	0.01455	0.00025	0.00653	0.00043	129.0	316.0	94.0	17.0	93.0	2.0	132.0	9.0	93	2
21TY265-051	0.05589	0.00246	0.35473	0.0154	0.04607	0.00059	0.02205	0.00141	448.0	74.0	308.0	12.0	290.0	4.0	441.0	28.0	290	4
21TY265-052	0.0555	0.00178	0.48926	0.01558	0.06399	0.00071	0.0252	0.00158	432.0	51.0	404.0	11.0	400.0	4.0	503.0	31.0	400	4
**21TY281**																		
2021TY281-001	0.04876	0.00896	0.14768	0.02707	0.02216	0.00041	0.00711	0.00044	136.0	311.0	140.0	24.0	141.0	3.0	143.0	9.0	141	3
2021TY281-002	0.11169	0.00144	4.81811	0.06594	0.31557	0.00367	0.06362	0.00328	1827.0	11.0	1788.0	12.0	1768.0	18.0	1247.0	62.0	1827	11
2021TY281-003	0.14633	0.00214	8.03205	0.12204	0.40146	0.00484	0.1161	0.00604	2303.0	12.0	2235.0	14.0	2176.0	22.0	2220.0	109.0	2303	12
2021TY281-004	0.09087	0.00167	2.91439	0.05443	0.23456	0.00286	0.0765	0.00395	1444.0	18.0	1386.0	14.0	1358.0	15.0	1490.0	74.0	1444	18
2021TY281-005	0.05042	0.01327	0.13518	0.03548	0.0196	0.00048	0.00755	0.00054	214.0	434.0	129.0	32.0	125.0	3.0	152.0	11.0	125	3
2021TY281-006	0.05133	0.00237	0.27105	0.01248	0.03861	0.0005	0.01388	0.00078	256.0	82.0	244.0	10.0	244.0	3.0	279.0	16.0	244	3
2021TY281-007	0.05513	0.00226	0.25026	0.01023	0.03317	0.00042	0.0318	0.00204	417.0	69.0	227.0	8.0	210.0	3.0	633.0	40.0	210	3
2021TY281-008	0.05168	0.00366	0.18598	0.0131	0.02628	0.00038	0.00771	0.00049	271.0	134.0	173.0	11.0	167.0	2.0	155.0	10.0	167	2
2021TY281-009	0.05526	0.00164	0.21822	0.00648	0.02884	0.00035	0.01379	0.00076	423.0	45.0	200.0	5.0	183.0	2.0	277.0	15.0	183	2

2021TY281-010	0.07352	0.00156	1.3263	0.02841	0.13174	0.00157	0.04238	0.00242	1028.0	25.0	857.0	12.0	798.0	9.0	839.0	47.0	798	9
2021TY281-011	0.0554	0.00369	0.20366	0.01349	0.02684	0.00039	0.00786	0.00043	428.0	123.0	188.0	11.0	171.0	2.0	158.0	9.0	171	2
2021TY281-012	0.09597	0.00187	3.39508	0.06688	0.2583	0.0032	0.07053	0.00378	1547.0	19.0	1503.0	15.0	1481.0	16.0	1378.0	71.0	1547	19
2021TY281-013	0.04632	0.01928	0.04744	0.0197	0.00748	0.00023	0.00306	0.00021	14.0	636.0	47.0	19.0	48.0	1.0	62.0	4.0	48	1
2021TY281-014	0.04926	0.00306	0.18712	0.01151	0.02773	0.00041	0.00855	0.00047	160.0	112.0	174.0	10.0	176.0	3.0	172.0	9.0	176	3
2021TY281-015	0.04773	0.00146	0.10447	0.00318	0.01597	0.0002	0.00518	0.00029	86.0	48.0	101.0	3.0	102.0	1.0	104.0	6.0	102	1
2021TY281-016	0.13562	0.0016	6.96921	0.08816	0.37496	0.00428	0.10825	0.00592	2172.0	10.0	2108.0	11.0	2053.0	20.0	2077.0	108.0	2172	10
2021TY281-017	0.05064	0.00265	0.19522	0.01018	0.02813	0.00036	0.00993	0.00056	224.0	97.0	181.0	9.0	179.0	2.0	200.0	11.0	179	2
2021TY281-018	0.05031	0.00256	0.05048	0.00256	0.00732	0.0001	0.00246	0.00014	209.0	92.0	50.0	2.0	47.0	0.6	50.0	3.0	47	1
2021TY281-019	0.05269	0.00258	0.2119	0.01032	0.02934	0.00039	0.00761	0.00043	315.0	87.0	195.0	9.0	186.0	2.0	153.0	9.0	186	2
2021TY281-020	0.06841	0.00282	1.02943	0.04228	0.10976	0.00145	0.03491	0.00192	881.0	63.0	719.0	21.0	671.0	8.0	694.0	37.0	671	8
2021TY281-021	0.09779	0.00163	3.56457	0.0608	0.26583	0.00317	0.08265	0.00454	1582.0	16.0	1542.0	14.0	1520.0	16.0	1605.0	85.0	1582	16
2021TY281-022	0.05199	0.00251	0.23856	0.01141	0.03346	0.00046	0.01099	0.00062	285.0	84.0	217.0	9.0	212.0	3.0	221.0	12.0	212	3
2021TY281-023	0.05522	0.00377	0.20853	0.01419	0.02753	0.00038	0.00792	0.00045	421.0	128.0	192.0	12.0	175.0	2.0	159.0	9.0	175	2
2021TY281-024	0.08087	0.00112	2.28031	0.033	0.20552	0.00236	0.06132	0.00342	1218.0	13.0	1206.0	10.0	1205.0	13.0	1203.0	65.0	1218	13
2021TY281-025	0.06173	0.00288	0.7738	0.03594	0.09137	0.00121	0.0286	0.00164	665.0	77.0	582.0	21.0	564.0	7.0	570.0	32.0	564	7
2021TY281-026	0.04967	0.00484	0.18227	0.01768	0.02674	0.00039	0.00755	0.00047	180.0	191.0	170.0	15.0	170.0	2.0	152.0	9.0	170	2
2021TY281-027	0.07987	0.00149	2.07073	0.03899	0.18893	0.00226	0.05134	0.00292	1194.0	19.0	1139.0	13.0	1116.0	12.0	1012.0	56.0	1194	19
2021TY281-028	0.26068	0.00283	22.81309	0.26861	0.63764	0.00722	0.16668	0.00944	3251.0	8.0	3219.0	11.0	3180.0	28.0	3116.0	164.0	3251	8
2021TY281-029	0.05735	0.0014	0.62903	0.01533	0.07988	0.00096	0.02242	0.00133	505.0	33.0	495.0	10.0	495.0	6.0	448.0	26.0	495	6
2021TY281-030	0.06507	0.00392	1.18061	0.07075	0.13212	0.0019	0.03845	0.00234	777.0	102.0	792.0	33.0	800.0	11.0	763.0	46.0	800	11
2021TY281-031	0.06488	0.01593	1.12629	0.27555	0.12641	0.00318	0.03064	0.00253	770.0	474.0	766.0	132.0	767.0	18.0	610.0	50.0	767	18
2021TY281-032	0.04743	0.00479	0.11628	0.01168	0.01785	0.00029	0.00415	0.00026	71.0	193.0	112.0	11.0	114.0	2.0	84.0	5.0	114	2
2021TY281-033	0.05299	0.00278	0.19934	0.0104	0.02738	0.00035	0.01188	0.00077	328.0	95.0	185.0	9.0	174.0	2.0	239.0	15.0	174	2
2021TY281-034	0.12125	0.00234	5.66292	0.10951	0.33982	0.00424	0.09364	0.00586	1975.0	18.0	1926.0	17.0	1886.0	20.0	1809.0	108.0	1975	18
2021TY281-035	0.06758	0.00244	1.31183	0.04486	0.14079	0.00161	0.04289	0.00047	856.0	77.0	851.0	20.0	849.0	9.0	849.0	9.0	849	9
2021TY281-036	0.06631	0.0014	1.24114	0.02634	0.13616	0.00162	0.04177	0.00256	816.0	25.0	819.0	12.0	823.0	9.0	827.0	50.0	823	9

2021TY281-037	0.06051	0.00228	0.64179	0.02411	0.07715	0.00096	0.01597	0.001	622.0	59.0	503.0	15.0	479.0	6.0	320.0	20.0	479	6
2021TY281-038	0.05608	0.00617	0.29843	0.03259	0.0386	0.00054	0.01202	0.00015	455.0	251.0	265.0	25.0	244.0	3.0	241.0	3.0	244	3
2021TY281-039	0.05502	0.00293	0.45905	0.02408	0.06067	0.00092	0.03276	0.00223	413.0	90.0	384.0	17.0	380.0	6.0	652.0	44.0	380	6
2021TY281-040	0.05798	0.00094	0.64024	0.01061	0.08026	0.00091	0.02225	0.00144	529.0	18.0	502.0	7.0	498.0	5.0	445.0	28.0	498	5
2021TY281-041	0.07167	0.00275	1.48373	0.0567	0.15045	0.0019	0.04557	0.00293	977.0	57.0	924.0	23.0	903.0	11.0	901.0	57.0	903	11
2021TY281-042	0.05091	0.003	0.20509	0.01194	0.02928	0.00045	0.01025	0.00069	237.0	106.0	189.0	10.0	186.0	3.0	206.0	14.0	186	3
2021TY281-043	0.05493	0.00448	0.45507	0.03681	0.0602	0.00099	0.01939	0.00132	409.0	153.0	381.0	26.0	377.0	6.0	388.0	26.0	377	6
2021TY281-044	0.05145	0.0019	0.23773	0.00874	0.03358	0.00042	0.01146	0.00077	261.0	61.0	217.0	7.0	213.0	3.0	230.0	15.0	213	3
2021TY281-045	0.05158	0.00199	0.19136	0.00731	0.02695	0.00035	0.00844	0.00056	267.0	64.0	178.0	6.0	171.0	2.0	170.0	11.0	171	2
2021TY281-046	0.05092	0.00721	0.19821	0.02795	0.02828	0.0005	0.00948	0.00081	237.0	280.0	184.0	24.0	180.0	3.0	191.0	16.0	180	3
2021TY281-047	0.06073	0.00396	0.52628	0.03366	0.06285	0.00082	0.01938	0.00022	630.0	145.0	429.0	22.0	393.0	5.0	388.0	4.0	393	5
2021TY281-048	0.11832	0.00128	5.69717	0.06641	0.34979	0.00387	0.09261	0.0061	1931.0	9.0	1931.0	10.0	1934.0	18.0	1790.0	113.0	1931	9
2021TY281-049	0.05783	0.00374	0.54105	0.03431	0.06786	0.00085	0.02105	0.00024	523.0	146.0	439.0	23.0	423.0	5.0	421.0	5.0	423	5
2021TY281-050	0.05211	0.00599	0.28699	0.03277	0.03999	0.00073	0.01096	0.0008	290.0	225.0	256.0	26.0	253.0	5.0	220.0	16.0	253	5
2021TY281-051	0.16871	0.00186	10.74957	0.12655	0.46256	0.00515	0.12325	0.00833	2545.0	9.0	2502.0	11.0	2451.0	23.0	2349.0	150.0	2545	9
2021TY281-052	0.09712	0.00127	3.74183	0.051	0.27967	0.00315	0.08091	0.00552	1570.0	12.0	1580.0	11.0	1590.0	16.0	1573.0	103.0	1570	12
2021TY281-053	0.05302	0.00385	0.29683	0.02145	0.04061	0.00056	0.01244	0.0009	330.0	140.0	264.0	17.0	257.0	3.0	250.0	18.0	257	3
2021TY281-054	0.06966	0.00113	1.17349	0.01942	0.12221	0.00139	0.0389	0.00275	918.0	17.0	788.0	9.0	743.0	8.0	771.0	54.0	743	8
2021TY281-055	0.04921	0.00552	0.16369	0.01812	0.02413	0.00053	0.00596	0.00047	158.0	207.0	154.0	16.0	154.0	3.0	120.0	9.0	154	3
2021TY281-056	0.05866	0.00289	0.6071	0.02976	0.07506	0.00097	0.02387	0.00174	555.0	85.0	482.0	19.0	467.0	6.0	477.0	34.0	467	6
2021TY281-057	0.05848	0.00331	0.63202	0.03564	0.07838	0.00101	0.02587	0.00186	548.0	101.0	497.0	22.0	486.0	6.0	516.0	37.0	486	6
2021TY281-058	0.05311	0.00388	0.19586	0.01424	0.02674	0.00037	0.00857	0.00062	333.0	141.0	182.0	12.0	170.0	2.0	172.0	12.0	170	2
2021TY281-059	0.05559	0.00268	0.50493	0.0242	0.06587	0.00087	0.01425	0.00108	436.0	83.0	415.0	16.0	411.0	5.0	286.0	22.0	411	5
2021TY281-060	0.05758	0.00105	0.59774	0.01113	0.07526	0.00085	0.02435	0.00177	514.0	22.0	476.0	7.0	468.0	5.0	486.0	35.0	468	5
2021TY281-061	0.04742	0.00386	0.17282	0.01395	0.02641	0.00042	0.00849	0.00065	70.0	148.0	162.0	12.0	168.0	3.0	171.0	13.0	168	3
2021TY281-062	0.05482	0.0054	0.29493	0.0289	0.03898	0.00061	0.01349	0.00113	405.0	193.0	262.0	23.0	247.0	4.0	271.0	23.0	247	4
2021TY281-063	0.04984	0.0045	0.26723	0.02403	0.03884	0.00055	0.01386	0.00112	188.0	176.0	240.0	19.0	246.0	3.0	278.0	22.0	246	3

2021TY281-064	0.05342	0.0018	0.45952	0.01539	0.06228	0.00078	0.01864	0.00146	347.0	53.0	384.0	11.0	389.0	5.0	373.0	29.0	389	5
2021TY281-065	0.05251	0.00392	0.28665	0.02127	0.03952	0.00058	0.01149	0.00092	308.0	143.0	256.0	17.0	250.0	4.0	231.0	18.0	250	4
2021TY281-066	0.05171	0.00184	0.24547	0.0087	0.03436	0.00041	0.0106	0.00085	273.0	59.0	223.0	7.0	218.0	3.0	213.0	17.0	218	3
2021TY281-067	0.05078	0.00305	0.26882	0.01601	0.03832	0.00054	0.01168	0.00095	231.0	111.0	242.0	13.0	242.0	3.0	235.0	19.0	242	3
2021TY281-068	0.07244	0.00143	1.62577	0.0322	0.16244	0.0019	0.04572	0.00361	998.0	22.0	980.0	12.0	970.0	11.0	904.0	70.0	970	11
2021TY281-069	0.05498	0.00092	0.52765	0.00894	0.06945	0.00078	0.02113	0.00169	411.0	19.0	430.0	6.0	433.0	5.0	423.0	33.0	433	5
2021TY281-070	0.07843	0.00311	1.588	0.06249	0.14651	0.00193	0.04362	0.00357	1158.0	57.0	966.0	25.0	881.0	11.0	863.0	69.0	881	11

Table S2 Calcite LA-ICP-MS U–Pb in-situ results

Sample	Intensity (cps)			Concentrations (ppm)			Isotope ratios			
	^{206}Pb	^{207}Pb	^{238}U	U	Th	Pb	$^{238}U/^{206}Pb$	2σ	$^{207}Pb/^{206}Pb$	2σ
**22TL025**										
22TL025-1	182696.7	147733.0	355645.6	2.775	10.258	5.465	1.881	0.063	0.818	0.008
22TL025-2	138311.9	113043.0	317524.3	2.480	6.981	4.202	2.202	0.042	0.820	0.006
22TL025-3	117359.7	95021.7	199575.4	1.560	6.028	3.545	1.627	0.031	0.812	0.007
22TL025-4	184405.8	149845.5	233417.5	1.827	7.718	5.597	1.234	0.039	0.813	0.005
22TL025-5	176704.4	142990.0	223455.7	1.750	9.563	5.369	1.232	0.033	0.816	0.006
22TL025-6	35306.0	28982.9	24595.7	0.193	0.610	1.097	0.667	0.013	0.824	0.008
22TL025-7	21799.1	17540.1	74362.4	0.584	0.284	0.652	3.286	0.073	0.807	0.009
22TL025-8	44700.2	36526.0	24738.0	0.194	0.832	1.374	0.529	0.017	0.820	0.008
22TL025-9	16277.8	13303.6	15762.0	0.124	0.153	0.497	0.951	0.042	0.819	0.009
22TL025-10	47229.9	38812.8	22257.7	0.176	1.157	1.464	0.457	0.019	0.824	0.007
22TL025-11	59836.0	49168.0	36164.6	0.287	1.596	1.886	0.576	0.010	0.823	0.006
22TL025-12	107479.8	87536.3	97848.3	0.776	2.995	3.356	0.876	0.029	0.817	0.007
22TL025-13	40594.1	32550.1	139633.7	1.109	4.038	1.254	3.409	0.365	0.804	0.008
22TL025-14	126290.5	103088.6	105863.0	0.841	3.024	3.921	0.863	0.052	0.818	0.006
22TL025-15	62248.8	50232.6	118636.7	0.943	3.179	1.926	1.825	0.039	0.809	0.007
22TL025-16	54412.8	43814.9	94225.2	0.749	2.442	1.692	1.658	0.029	0.808	0.007
22TL025-17	107556.1	87474.8	142628.2	1.135	3.819	3.338	1.677	0.129	0.817	0.007
22TL025-18	57679.8	46511.3	114929.6	0.915	3.286	1.785	1.891	0.058	0.809	0.007
22TL025-19	64699.8	53064.2	85644.0	0.683	2.233	2.040	1.278	0.030	0.822	0.007
22TL025-20	47082.7	38408.0	63666.6	0.513	1.596	1.475	1.300	0.025	0.819	0.008
22TL025-21	46298.7	37717.8	105786.0	0.853	2.762	1.458	2.195	0.035	0.819	0.007
22TL025-22	26232.7	21332.2	41626.9	0.336	0.791	0.822	1.554	0.053	0.820	0.010
22TL025-23	54664.2	44595.9	99382.0	0.802	2.540	1.708	1.733	0.050	0.821	0.007
22TL025-24	40704.5	31255.9	295319.1	2.386	1.952	1.218	7.080	0.256	0.769	0.008
22TL025-25	70107.0	56684.0	274528.1	2.220	2.669	2.197	3.853	0.122	0.811	0.007
22TL025-26	65018.1	52087.3	226699.6	1.834	2.535	2.008	3.403	0.077	0.804	0.007
22TL025-27	51878.6	41579.8	257624.8	2.086	1.767	1.583	4.840	0.111	0.798	0.009
22TL025-28	101313.5	82086.7	137406.0	1.113	5.300	3.167	1.310	0.024	0.813	0.007
22TL025-29	91771.4	73790.7	184701.4	1.496	6.568	2.854	1.941	0.035	0.806	0.007
22TL025-30	194482.0	160800.4	107431.4	0.867	7.589	6.203	0.532	0.015	0.827	0.006
22TL025-31	140066.7	114588.0	135929.2	1.096	9.396	4.422	0.927	0.016	0.820	0.006
22TL025-32	35275.3	27983.4	191652.8	1.544	4.415	1.088	5.380	0.241	0.795	0.008
22TL025-33	60172.1	47946.3	251021.5	2.020	6.078	1.874	4.120	0.143	0.797	0.008
22TL025-34	32041.3	24895.8	283827.0	2.282	2.054	0.952	8.574	0.209	0.778	0.008
22TL025-35	39957.6	31705.6	258096.0	2.074	2.222	1.233	6.210	0.166	0.789	0.008
22TL025-36	38087.7	30097.1	246416.3	1.978	2.827	1.163	8.213	0.993	0.779	0.010
22TL025-37	65947.3	52810.4	201589.6	1.617	3.461	2.057	3.237	0.192	0.802	0.007
22TL025-38	95578.2	78599.5	103130.4	0.826	1.933	3.021	1.011	0.063	0.825	0.007
22TL025-39	72266.9	58946.6	35861.2	0.287	0.589	2.250	0.523	0.039	0.820	0.007
22TL025-40	189516.7	154673.7	239255.1	1.906	6.284	6.013	1.199	0.022	0.818	0.005
22TL025-41	196330.3	160647.2	255831.2	2.038	6.625	6.168	1.237	0.020	0.820	0.006
22TL025-42	159303.4	129951.8	191370.5	1.525	7.100	5.057	1.143	0.015	0.819	0.006
22TL025-43	196343.6	160605.3	248986.0	1.983	9.024	6.193	1.211	0.025	0.818	0.006
22TL025-44	185189.1	151801.8	231205.8	1.842	7.567	5.860	1.189	0.020	0.822	0.006
22TL025-45	211846.6	173589.3	385630.4	3.072	9.365	6.696	1.740	0.046	0.820	0.006

22TL025-46	192405.6	157326.3	341616.1	2.722	10.338	6.080	1.682	0.026	0.820	0.006
22TL025-47	205422.9	167241.7	208303.6	1.660	9.864	6.471	0.958	0.014	0.815	0.006
22TL025-48	145880.9	118598.2	150201.8	1.197	9.105	4.656	0.983	0.021	0.815	0.006
22TL025-49	194174.9	158933.1	353029.7	2.815	10.666	6.199	1.734	0.035	0.821	0.005
22TL025-50	106642.9	87084.0	123207.8	0.987	8.388	3.395	1.094	0.016	0.818	0.006
22TL025-51	86921.7	70729.8	116290.1	0.932	6.788	2.770	1.262	0.027	0.820	0.006
22TL025-52	103186.5	83852.1	152729.2	1.224	9.549	3.290	1.398	0.023	0.816	0.007
22TL025-53	123581.7	100267.4	203123.8	1.629	11.985	3.919	1.557	0.030	0.814	0.006
22TL025-54	65588.4	53223.9	145728.7	1.169	7.022	2.094	2.105	0.086	0.813	0.007
22TL025-55	91495.6	73852.0	160657.3	1.289	7.050	2.865	1.659	0.025	0.809	0.007
22TL025-56	76120.3	61726.1	128525.4	1.032	7.381	2.408	1.602	0.041	0.813	0.007
22TL025-57	112890.7	92241.9	113683.1	0.913	7.046	3.613	0.950	0.016	0.819	0.007
22TL025-58	92552.8	74829.5	145418.1	1.168	5.822	2.937	1.494	0.035	0.811	0.006
22TL025-59	110531.5	89495.7	137072.3	1.101	6.059	3.503	1.196	0.041	0.811	0.006
22TL025-60	98125.0	79753.9	143281.1	1.150	5.417	3.141	1.374	0.026	0.815	0.006
22TL025-61	116239.6	95014.1	123691.5	0.992	4.575	3.751	0.999	0.018	0.819	0.006
22TL025-62	100773.0	81811.1	111954.5	0.898	4.182	3.180	1.047	0.029	0.813	0.006
22TL025-63	77557.7	62379.1	151728.2	1.216	4.066	2.468	1.855	0.043	0.809	0.007
22TL025-64	80101.5	65118.2	160922.6	1.289	4.556	2.552	1.893	0.032	0.812	0.007
22TL025-65	73955.0	59794.0	143999.8	1.153	3.918	2.345	1.800	0.045	0.809	0.006
22TL025-66	125011.8	101686.9	172170.4	1.378	11.580	4.002	1.289	0.019	0.817	0.006
22TL025-67	140766.0	114037.3	198257.4	1.587	16.500	4.510	1.321	0.021	0.816	0.006
22TL025-68	112818.2	91659.3	145248.1	1.162	12.637	3.622	1.200	0.017	0.811	0.005
22TL025-69	163060.9	133389.6	199380.9	1.595	13.398	5.259	1.145	0.019	0.820	0.006
22TL025-70	79371.0	64291.3	135235.6	1.082	11.152	2.572	1.583	0.032	0.811	0.007
22TL025-71	75013.8	60458.4	143531.6	1.149	11.273	2.421	1.783	0.037	0.809	0.007
22TL025-72	214697.5	175253.9	310450.2	2.485	16.265	6.974	1.342	0.031	0.818	0.006
22TL025-73	93991.7	75939.5	159464.5	1.277	11.838	3.034	1.580	0.032	0.810	0.006
22TL025-74	449485.5	368239.0	405449.9	3.249	11.430	14.470	0.845	0.027	0.822	0.005
22TL025-75	342605.5	280123.5	476177.0	3.818	16.793	11.082	1.297	0.020	0.820	0.005
22TL025-76	304714.9	250212.5	518732.1	4.161	14.818	9.968	1.596	0.054	0.823	0.005
22TL025-77	465613.1	379612.1	718623.2	5.768	13.692	15.061	1.443	0.034	0.818	0.005
22TL025-78	126174.9	102568.1	198597.0	1.595	5.963	4.077	1.466	0.039	0.815	0.006
22TL025-79	138656.7	112657.8	178324.1	1.433	6.813	4.517	1.196	0.023	0.816	0.006
22TL025-80	138992.6	112969.1	215147.3	1.747	10.502	4.592	1.441	0.025	0.816	0.006
22TL025-81	157162.5	129420.2	238310.0	1.937	9.412	5.214	1.408	0.028	0.825	0.006
22TL025-82	150633.9	123182.7	115719.4	0.941	4.995	5.023	0.714	0.013	0.821	0.006
22TL025-83	177765.4	145814.4	134205.2	1.092	4.097	5.816	0.732	0.038	0.821	0.006
22TL025-84	209819.4	170955.2	154100.9	1.255	4.897	6.869	0.683	0.014	0.816	0.006
22TL025-85	187273.6	153002.3	142046.2	1.158	4.598	6.169	0.708	0.015	0.819	0.006
22TL025-86	198698.0	161866.6	224051.7	1.828	5.923	6.546	1.061	0.027	0.816	0.006
22TL025-87	180314.7	147697.9	262209.4	2.140	5.656	5.937	1.352	0.023	0.821	0.006
22TL025-88	221997.6	181677.2	207047.8	1.690	21.653	7.397	0.862	0.012	0.818	0.006
22TL025-89	229902.9	187154.1	194798.3	1.591	22.161	7.598	0.791	0.021	0.817	0.006
22TL025-90	339960.6	278970.5	271851.8	2.213	27.766	11.242	0.754	0.023	0.823	0.005
22TL025-91	262962.1	215054.1	268025.3	2.180	26.953	8.770	0.944	0.017	0.820	0.006
22TL025-92	243134.5	198164.7	453455.4	3.685	19.895	8.016	1.720	0.033	0.817	0.006
22TL025-93	200078.3	164408.0	107334.5	0.872	17.436	6.683	0.503	0.013	0.825	0.005
22TL025-94	143968.0	117923.2	88625.0	0.719	16.064	4.826	0.569	0.008	0.821	0.006
22TL025-95	171589.1	140467.7	77351.8	0.627	23.640	5.718	0.418	0.010	0.821	0.006

22TL025-96	243402.5	199209.4	93706.7	0.759	23.006	8.047	0.358	0.007	0.822	0.006
22TL025-97	251590.3	206714.9	108062.2	0.874	21.749	8.368	0.397	0.010	0.823	0.006
22TL025-98	139483.9	113488.4	166664.6	1.347	8.629	4.590	1.106	0.019	0.817	0.006
22TL025-99	214335.1	175959.3	251625.8	2.031	7.632	7.050	1.088	0.039	0.823	0.006
**21TY222**										
21TY222-01	81033.8	65052.0	161027.0	1.626	2.105	2.694	0.802	0.005	1.983	0.033
21TY222-02	81346.6	64811.0	161313.0	1.641	1.896	2.632	0.799	0.006	2.036	0.034
21TY222-03	83698.8	66422.3	166031.7	1.699	3.163	2.647	0.793	0.006	2.112	0.069
21TY222-04	60651.4	47712.6	120375.0	1.829	1.507	1.877	0.787	0.005	3.318	0.142
21TY222-05	63911.0	51049.9	128289.7	1.421	1.069	1.965	0.800	0.006	2.409	0.043
21TY222-06	86777.7	69407.1	174565.4	1.582	1.683	2.638	0.803	0.005	2.002	0.031
21TY222-07	89109.7	71810.6	178643.0	1.692	1.940	2.678	0.804	0.005	2.139	0.106
21TY222-08	81132.2	64495.4	160746.7	1.465	1.524	2.408	0.796	0.005	2.005	0.042
21TY222-09	91041.9	72996.7	182820.1	1.378	1.628	2.759	0.801	0.005	1.670	0.046
21TY222-10	94233.3	75804.7	189026.7	1.594	2.119	2.904	0.804	0.005	1.834	0.032
21TY222-11	103451.9	83176.4	207356.6	1.699	1.968	3.836	0.804	0.006	1.498	0.035
21TY222-12	82383.1	65447.6	163713.3	1.778	1.775	3.011	0.793	0.006	1.958	0.025
21TY222-13	84403.0	67592.1	167934.3	1.688	1.868	3.061	0.800	0.005	1.858	0.040
21TY222-14	81140.2	64860.4	162454.4	1.597	1.864	2.926	0.798	0.006	1.829	0.035
21TY222-15	75282.7	60139.1	150599.5	1.640	2.026	2.676	0.797	0.005	2.069	0.040
21TY222-16	84431.8	67871.3	169159.8	1.653	1.971	2.962	0.800	0.005	1.886	0.032
21TY222-17	89462.3	71460.8	178078.8	1.694	2.077	3.072	0.799	0.005	1.846	0.028
21TY222-18	92105.2	73148.0	185858.1	1.775	1.900	3.160	0.799	0.004	1.923	0.041
21TY222-19	92840.0	75110.7	186149.5	1.716	1.744	3.122	0.807	0.005	1.862	0.046
21TY222-20	85581.2	68161.4	169850.6	1.940	1.869	2.815	0.797	0.005	2.297	0.049
21TY222-21	86365.1	68745.4	173603.6	2.025	1.891	2.743	0.796	0.006	2.441	0.045
21TY222-22	88917.7	70759.7	175774.9	2.152	2.262	2.773	0.795	0.005	2.569	0.080
21TY222-23	86083.5	68393.2	171054.1	2.081	2.129	2.697	0.792	0.005	2.522	0.053
21TY222-24	86120.1	68504.2	171453.8	1.959	1.715	2.704	0.797	0.004	2.388	0.034
21TY222-25	98326.3	79543.2	199464.0	1.937	1.868	3.149	0.800	0.004	2.080	0.062
21TY222-26	95249.4	76288.8	190452.1	2.027	1.853	3.013	0.800	0.005	2.233	0.059
21TY222-27	66659.0	53299.6	132986.9	1.875	1.567	2.110	0.798	0.005	2.940	0.046
21TY222-28	70059.2	56018.0	140514.1	1.918	1.632	2.238	0.798	0.006	2.835	0.040
21TY222-29	162239.0	130547.2	324785.6	2.795	2.516	5.200	0.803	0.004	1.789	0.035
21TY222-30	116677.4	93512.5	231196.8	2.478	2.206	3.726	0.800	0.004	2.189	0.036
21TY222-31	131583.2	105318.8	261872.1	2.738	2.524	4.489	0.798	0.004	2.014	0.034
21TY222-32	140942.9	112726.5	281336.0	3.203	3.127	4.837	0.800	0.004	2.158	0.030
21TY222-33	84476.4	67551.8	167688.9	1.962	2.252	2.887	0.797	0.005	2.264	0.074
21TY222-34	81731.0	65095.9	161674.6	1.837	1.688	2.784	0.796	0.006	2.179	0.036
21TY222-35	81704.4	65272.1	163229.4	1.808	1.860	2.807	0.798	0.005	2.137	0.035
21TY222-36	87574.1	69878.2	174069.6	1.863	1.851	2.985	0.796	0.004	2.065	0.043
21TY222-37	87892.1	70158.8	175323.6	2.186	2.276	2.992	0.796	0.005	2.476	0.079
21TY222-38	90827.7	72383.6	181374.9	2.193	2.525	3.076	0.794	0.005	2.416	0.071
21TY222-39	90004.5	71953.4	180335.5	2.108	2.222	3.033	0.799	0.004	2.333	0.047
21TY222-40	87062.2	69908.0	172903.8	2.042	1.970	2.878	0.802	0.005	2.347	0.034
21TY222-41	83041.0	66820.7	166272.1	1.636	1.900	2.481	0.803	0.005	2.179	0.048
21TY222-42	82604.1	65516.9	162294.9	1.740	1.814	2.407	0.791	0.006	2.368	0.065
21TY222-43	74253.9	59637.7	147481.9	1.679	1.578	2.179	0.802	0.005	2.507	0.029
21TY222-44	79444.6	63437.8	159159.2	1.755	2.061	2.350	0.798	0.005	2.482	0.042
21TY222-45	98019.3	78105.9	195671.4	1.641	2.270	2.894	0.795	0.005	1.867	0.029

21TY222-46	88923.7	70928.5	176823.8	1.450	1.901	2.628	0.798	0.005	1.815	0.034
21TY222-47	87777.5	70124.5	174285.4	1.505	1.790	2.613	0.797	0.005	1.915	0.051
21TY222-48	94264.6	75151.2	188533.7	1.400	1.886	2.861	0.796	0.005	1.637	0.043
21TY222-49	84842.7	67686.5	169847.8	1.665	2.131	2.621	0.795	0.005	2.104	0.059
21TY222-50	79710.1	63398.6	159028.6	1.665	1.852	2.510	0.795	0.005	2.187	0.047
21TY222-51	48128.4	38434.4	94512.8	1.597	1.340	1.846	0.801	0.013	2.848	0.088
21TY222-52	57989.0	45074.5	111258.5	2.211	1.796	2.175	0.786	0.009	3.261	0.062
21TY222-53	81040.0	64947.2	158669.5	3.046	1.990	3.104	0.798	0.012	3.158	0.091
21TY222-54	91057.0	72901.5	179795.6	1.943	2.726	3.521	0.804	0.006	1.813	0.039
21TY222-55	122526.2	99882.1	239821.5	2.464	3.521	4.699	0.814	0.014	1.687	0.064
21TY222-56	66668.1	52004.6	127783.6	3.475	1.818	2.506	0.777	0.010	4.442	0.117
21TY222-57	54651.1	43296.1	107116.6	2.226	1.857	2.102	0.796	0.008	3.471	0.086
21TY222-58	58252.6	45640.4	112903.4	2.600	2.083	2.218	0.788	0.007	3.837	0.091
21TY222-59	50693.9	39341.0	96531.2	2.527	1.873	1.897	0.783	0.007	4.293	0.072
21TY222-60	66346.9	51842.6	129716.5	3.096	1.969	2.552	0.786	0.010	3.992	0.092
21TY222-61	75694.7	60186.1	147629.0	2.727	1.775	2.933	0.793	0.011	3.017	0.109
21TY222-62	72161.0	57034.5	140759.4	3.040	2.460	2.799	0.793	0.008	3.541	0.078
21TY222-63	64787.5	50292.9	125811.1	2.558	2.147	2.504	0.779	0.010	3.330	0.085
21TY222-64	54178.1	42686.3	105544.6	2.208	1.821	2.102	0.789	0.008	3.435	0.105
21TY222-65	56901.7	44818.2	109309.9	2.264	1.796	2.179	0.789	0.009	3.374	0.086
21TY222-66	41642.5	32603.0	79999.9	1.730	1.257	1.596	0.788	0.014	3.502	0.098
21TY222-67	52639.3	40906.3	102352.7	2.437	1.994	2.043	0.782	0.007	3.875	0.089
21TY222-68	55375.3	43675.8	107531.7	2.264	2.001	2.148	0.793	0.007	3.439	0.091
21TY222-69	53433.4	41605.7	103336.6	1.994	1.779	2.066	0.787	0.007	3.190	0.068
21TY222-70	67873.9	52574.6	131641.3	2.430	2.154	2.634	0.784	0.012	3.022	0.128
21TY222-71	63134.7	50104.8	125364.3	2.716	2.452	2.534	0.799	0.008	3.548	0.088
21TY222-72	65285.2	51782.3	128462.0	2.365	2.151	2.599	0.796	0.010	2.998	0.098
21TY222-73	75902.9	59747.6	148540.3	2.156	2.359	3.008	0.793	0.010	2.343	0.036
21TY222-74	77960.4	61810.2	154233.6	2.465	3.343	3.125	0.798	0.008	2.604	0.208
21TY222-75	77533.4	60981.1	150315.3	2.555	2.413	3.048	0.794	0.007	2.736	0.068
21TY222-76	79186.6	62232.0	155535.8	2.390	2.440	3.157	0.791	0.007	2.495	0.045
21TY222-77	75317.9	59657.8	148232.0	2.334	2.363	3.011	0.798	0.006	2.567	0.051
21TY222-78	89734.7	71472.6	177825.4	2.390	2.860	3.615	0.799	0.006	2.207	0.055
21TY222-79	85469.8	67331.3	169039.4	2.389	2.747	3.439	0.794	0.006	2.355	0.081
21TY222-80	80886.2	63690.8	160286.6	2.486	4.187	3.263	0.794	0.006	2.537	0.078
21TY222-81	74866.1	58910.8	147232.8	2.310	2.181	3.029	0.795	0.007	2.524	0.056
21TY222-82	79112.4	62005.2	154664.7	2.522	2.403	3.185	0.790	0.006	2.630	0.067
21TY222-83	93829.2	74174.9	185548.4	2.408	2.020	3.823	0.798	0.006	2.181	0.073
21TY222-84	68104.8	53252.8	132932.4	2.259	2.042	2.742	0.792	0.006	2.708	0.049
21TY222-85	67111.0	52923.5	130254.4	2.443	1.921	2.688	0.794	0.008	2.975	0.081
21TY222-86	77559.6	61615.3	154418.0	2.377	2.289	3.190	0.796	0.007	2.499	0.036
21TY222-87	86501.3	68483.4	168457.7	2.290	2.612	3.483	0.799	0.006	2.150	0.046
21TY222-88	82696.9	66042.3	163539.0	2.270	2.827	3.386	0.805	0.006	2.227	0.049
21TY222-89	82442.9	65723.6	163695.1	2.009	2.684	3.392	0.804	0.008	1.990	0.052
21TY222-90	72981.8	57533.9	143194.3	1.899	2.345	2.999	0.792	0.008	2.083	0.048
21TY222-91	78762.4	62899.5	154203.6	1.806	2.697	3.232	0.806	0.007	1.832	0.045
21TY222-92	86687.5	68530.3	169490.2	1.945	2.375	3.556	0.797	0.007	1.810	0.027
21TY222-93	47666.2	37586.8	93159.9	1.362	1.905	1.956	0.798	0.007	2.297	0.054
21TY222-94	74511.3	58949.5	144976.8	2.034	2.800	3.046	0.798	0.006	2.208	0.068
21TY222-95	95921.1	75801.7	189209.3	2.681	3.287	3.979	0.797	0.007	2.223	0.034

21TY222-96	94783.9	74805.9	185465.7	2.777	3.083	3.903	0.794	0.008	2.353	0.068
21TY222-97	98595.7	78466.2	192352.0	2.251	2.628	4.052	0.803	0.009	1.789	0.031
21TY222-98	89928.8	71410.7	173824.8	2.587	2.040	3.667	0.801	0.011	2.217	0.053

Table S3 Clumped isotopic results of three limestone samples

Sample Name	$\delta^{13}C$ ‰ VPDB	$\delta^{18}O$ ‰ VPDB	δ_{47} vs WG (PBL)	Δ_{47} vs WG (PBL)	δ_{48} vs WG (PBL)	Δ_{48} vs WG (PBL)	$\Delta_{47\text{-ARF}}$	$\Delta_{47,\text{ ARF-AC}}$	STF-$\Delta_{47,\text{ ARF-AC}}$	Δ_{48} offset	Anderson et al., 2021	T(°C)	Average (°C)	1σ
21TY219-R1	−0.08	−11.04	38.938	−0.360	35.848	0.494	0.573	0.661	0.649	0.475	310.13	37.0	36.0	3.2
21TY219-R2	−0.08	−11.03	38.939	−0.366	35.973	0.602	0.567	0.655	0.642	0.583	312.46	39.3		
21TY219-R3	−0.08	−10.99	39.004	−0.347	37.064	1.568	0.587	0.675	0.663	1.549	304.89	31.7		
21TY222-1-R1	−0.46	−11.53	38.099	−0.275	34.369	0.099	0.582	0.670	0.670	0.148	302.14	29.0	33.5	3.5
21TY222-1-R2	−0.46	−11.54	38.082	−0.287	34.497	0.231	0.568	0.656	0.656	0.279	307.20	34.1		
21TY222-1-R3	−0.47	−11.54	38.070	−0.294	34.465	0.198	0.559	0.647	0.647	0.246	310.59	37.4		
22TY222-2-R1	−0.31	−10.98	38.770	−0.360	36.059	0.575	0.573	0.661	0.649	0.556	310.13	37.0	37.9	0.8
21TY222-2-R2	−0.33	−11.01	38.714	−0.364	35.991	0.584	0.569	0.657	0.644	0.565	311.68	38.5		
21TY222-2-R3	−0.32	−10.99	38.740	−0.365	41.470	5.835	0.568	0.656	0.643	5.817	312.07	38.9		
21TY222-2-R4	−0.32	−11.00	38.739	−0.362	37.781	2.281	0.572	0.660	0.648	2.262	310.52	37.4		

Table S4 Stable isotopic results of three limestone samples		
Sample Name	$\delta^{13}C$ ‰ (VPDB)	$\delta^{18}O$ ‰ (VPDB)
21TY219	−0.39	−11.79
21TY222-1	−0.46	−11.54
21TY222-2	−0.32	−11.00

Table S5 Stable isotopic paleoelevation reconstruction

Sample Name	Description	$\delta^{18}O$(‰) VPDB	$\delta^{18}O$(‰) VSMOW	T (K)	T (°C)	lnα(c-p)	α	$\delta^{18}O_{cw}$ (‰)	corrected $\delta^{18}O_{cw}$ (‰)	Paleoelevation (m)	1σ+(m)	1σ−(m)
21TY219	limestone	−11.79	18.76	309.2	36.0	0.03	1.0	−7.29	−6.09	803	519	−527
21TY222-1	limestone	−11.54	19.01	306.7	33.5	0.03	1.0	−7.51	−6.31	947	557	−566
21TY222-2	limestone	−11.00	19.57	311.0	37.8	0.03	1.0	−6.16	−4.96	50	237	−239
Average	/	−11.44	19.11	308.9	35.8	0.03	1.0	−6.99	−5.79	604	198	−426

Table S6 Climate Leaf Analysis Multivariate Program (CLAMP) scoresheet for the Luolong Flora

Species Morphotypes	Lamina		Margin Character States										Size Character States									Apex Character States				Base Character States			Length to Width Character States					Shape Character States		
	Unlobed	Lobed	No Teeth	Teeth	Teeth Regular	Teeth Irregular	Teeth Close	Teeth Distant	Teeth Round	Teeth Acute	Teeth Compound	Compound<50%	Nanophyll	Leptophyll I	Leptophyll II	Microphyll I	Microphyll II	Microphyll III	Mesophyll I	Mesophyll II	Mesophyll III	Emarginate	Round	Acute	Attenuate	Cordate	Round	Acute	L:W <1:1	L:W 1-2:1	L:W 2-3:1	L:W 3-4:1	L:W >4:1	Obovate	Elliptic	Ovate
OTU 1		1		1	1		1			1					1	1								1			1	1					1		1	
OTU 2	1			1		1		1		1						1												1			1					1
OTU 3	1			1	1		1		1									1							1		1					1				1
OTU 4	1		1																	1				1				1					1		1	
OTU 5	1		1	1	1		1			1							1											1					1		1	
OTU 6	1		1													1	1							1				1			1	1			1	
OTU 7	1			1	1		1			1						1								1				1				1				1
OTU 8	1		1																								1	1							1	
OTU 9	1		1														1											1							1	
OTU 10	1		1															1										1			1					1
OTU 11	1		1													1								1			1				1					1
OTU 12	1			1	1		1			1						1	1	1					1				1			1	1				1	
OTU 13	1		1													1	1	1						1			1	1		1					1	
OTU 14	1		1													1	1										1	1		1	1				1	
OTU 15	1		1														1														1					1
OTU 16	1		1															1							1	1				1					1	
OTU 17	1			1	1		1			1						1										1				1						1
OTU 18	1			1		1	1			1		1															1									
OTU 19	1			1		1	1		1			1			1								1				1		1						1	
OTU 20	1			1		1	1			1						1								1			1			1						1
OTU 21	1		1													1	1	1						1				1				1				1
OTU 22	1		1													1	1							1			1	1			1	1			1	

OTU 23	1		1													1								1				1			1	1			1	
OTU 24	1			1	1			1	1							1								1				1			1					1
OTU 25	1			1	1		1		1			1				1								1				1					1			1
OTU 26	1		1														1							1			1				1				1	1
OTU 27	1			1	1		1		1	1						1	1							1			1	1					1			1
OTU 28	1			1	1			1		1		1				1	1	1						1			1	1					1		1	1
OTU 29	1		1														1	1						1				1					1		1	
OTU 30	1		1	1	1			1		1						1								1				1					1		1	
OTU 31	1			1	1		1		1	1		1						1	1						1		1			1	1					1
OTU 32	1		1																						1											1
OTU 33	1			1	1		1			1						1								1			1						1			1
OTU 34	1			1	1		1			1						1								1			1						1			1
OTU 35	1			1	1			1		1						1								1				1					1		1	
OTU 36	1			1	1		1			1							1							1			1					1				1
OTU 37	1		1													1								1				1				1				1
OTU 38	1			1		1		1		1		1						1						1			1				1					1
OTU 39		1	1																								1								1	
OTU 40		1	1																	1								1					1		1	
OTU 41		1	1															1					1			1			1					1		
OTU 42	1		1														1							1				1				1				1
OTU 43	1		1													1									1			1			1					1
OTU 44	1		1													1							1					1			1				1	
OTU 45	1		1													1							1				1			1				1		
OTU 46	1		1													1											1			1					1	
OTU 47	1			1	1			1		1		1				1								1			1						1			1
OTU 48	1		1													1								1			1				1					1
OTU 49	1		1															1						1			1				1					1

**OTU 50**		**1**		**1**		**1**	**1**			**1**	**1**						**1**							**1**		**1**				**1**						**1**
**OTU 51**	**1**		**1**																		**1**															

Table S7 CLAMP scores of leaf fossil physiognomy in the Luolong Flora

Dissection	Lobed	9.8
Margin Character States	No Teeth	56.9
	Teeth Regular	31.4
	Teeth Close	30.4
	Teeth Round	9.8
	Teeth Acute	33.3
	Teeth Compound	2.0
Size Character States	Nanophyll	0.0
	Leptophyll I	0.0
	Leptophyll II	3.2
	Microphyll I	48.6
	Microphyll II	23.0
	Microphyll III	17.7
	Mesophyll I	1.1
	Mesophyll II	4.3
	Mesophyll III	2.1
Apex Character States	Apex Emarg.	0.0
	Apex Round	13.2
	Apex Acute	73.7
	Apex Atten.	13.2
Base Character States	Base Cordate	8.3
	Base Round	44.8
	Base Acute	46.9
Length to Wideth Character States	L:W<1:1	4.4
	L:W 1-2:1	18.9
	L:W 2-3:1	31.1
	L:W 3-4:1	16.7
	L:W>4:1	28.9
Shape Character States	Obovate	4.1
	Elliptic	42.9
	Ovate	53.1
Completeness	0.84	

Table S8 Climate parameters of the Luolong Flora

Assemblage	MAAT (°C)	WMMT (°C)	CMMT (°C)	LGS (Months)	GSP (mm)	X3WET (mm)	X3DRY (mm)	SH (g/kg)	ENTHAL (kJ/kg)	VPD.Ann (hPa)
Luolong	13.0	24.9	2.0	8.2	1481	728	217	6.2	311.0	6.9
Uncertainties	2.4	2.9	3.5	1.1	643	400	98	1.8	8.4	2.4

Assemblage	VPD.Win (hPa)	VPD.Spr (hPa)	VPD.Sum (hPa)	VPD_Aut (hPa)	MINT.Wrm (°C)	MAXT.Cld (°C)	GDD5_div1000 /	PET.Ann_div10 (mm/dayx10^{-1})	PET.Wrm (mm/dayx10^{-1})	PET.Cld (mm/dayx10^{-1})
Luolong	2.8	5.0	11.5	8.0	20.7	7.5	57.5	104.6	146.5	25.2
Uncertainties	1.5	4.0	3.5	2.0	2.9	3.5	10.6	16.6	24.5	14.0

MAAT, mean annual air temperature; WMMT, warm month mean temperature; CMMT, cold month mean temperature;
LGS, length of the growing season; GSP, growing season precipitation;
X3WET, precipitation in the three consecutive wettest months; X3DRY, precipitation in the three consecutive driest months;
SH, mean annual specific humidity; ENTHAL, mean annual moist enthalpy; VPD. Ann, mean annual vapor pressure deficit;
VPD. Win, mean vapor pressure deficit during winter (DJF); VPD. Spr, mean vapor pressure deficit during spring (MAM);
VPD. Sum, mean vapor pressure deficit during summer (JJA); VPD. Aut, mean vapor pressure deficit during fall (SON);
MINT. Wrm, mean minimum temperature of the warmest month; MAXT. Cld, mean maximum temperature of the coldest month mean;
GDD5_div1000, growing degree days above 5°C; PET.Ann_div10, mean annual potential evapotranspiration;
PET. Wrm, mean evapotranspiration during the warmest month; PET. Cld, mean potential evapotranspiration during the coldest month.

7. References

1. Sláma, J., Košler, J., Condon, DJ *et al.* Plešovice zircon−a new natural reference material for U−Pb and Hf isotopic microanalysis. *Chem Geol* 2008; **249**: 1−35.
2. Wiedenbeck M, Alle P, Corfu F *et al.* 3 natural zircon standards for U−Th−Pb, Lu−Hf, Trace-element and REE analysis. *Geostand Newsl* 1995; **19**: 1−23.
3. Andersen T. Correction of common lead in U–Pb analyses that do not report ^{204}Pb. *Chem Geol* 2002; **192**: 59−79.
4. Wu S, Yang Y, Roberts NMW *et al.* In situ calcite U−Pb geochronology by high-sensitivity single-collector LA-SF-ICP-MS (in Chinese). *Sci China Earth Sci* 2022; **65**: 1146−60.
5. Wu S, Wörner G, Jochum KP *et al.* The Preparation and Preliminary Characterisation of Three Synthetic Andesite Reference Glass Materials (ARM-1, ARM-2, ARM-3) for In Situ Microanalysis. *Geostand Geoanal Res* 2019; **43**: 567−84.
6. Roberts NMW, Rasbury ET, Parrish RR *et al.* A calcite reference material for LA-ICP-MS U−Pb geochronology. *Geochem Geophy Geosy* 2017; **18**: 2807−14.
7. Zhang L-L, Zhu D-C, Xie J-C *et al.* TARIM calcite: a potential reference material for laser ICP-MS in situ calcite U–Pb dating. *J Anal At Spectrom* 2023; **38**: 2302−12.
8. Dennis KJ, Affek HP, Passey BH *et al.* Defining an absolute reference frame for 'clumped' isotope studies of CO_2. *Geochim Cosmochim Acta* 2011; **75**: 7117−31.
9. Chang B, Defliese WF, Li C *et al.* Effects of different constants and standards on the reproducibility of carbonate clumped isotope (Δ_{47}) measurements: Insights from a long-term dataset. *Rapid Commun Mass Spectrom* 2020; **34**: e8678.
10. John CM and Bowen D. Community software for challenging isotope analysis: First applications of 'Easotope' to clumped isotopes. *Rapid Commun Mass Spectrom* 2016; **30**: 2285−300.
11. Fick SE and Hijmans RJ. WorldClim 2: new 1-km spatial resolution climate surfaces for global land areas. *Int J Climatol* 2017; **37**: 4302−15.
12. Forest CE, Molnar P, Emanuel KA. Palaeoaltimetry from energy conservation principles. *Nature* 1995; **374**: 347−50.
13. Henkes GA, Passey BH, Grossman EL *et al.* Temperature limits for preservation of primary calcite clumped isotope paleotemperatures. *Geochim Cosmochim Acta* 2014; **139**: 362−82.
14. Quade J, Eiler J, Daëron M *et al.* The clumped isotope geothermometer in soil and paleosol carbonate. *Geochim Cosmochim Acta* 2013; **105**: 92−107.
15. Currie BS, Rowley DB, Tabor NJ. Middle Miocene paleoaltimetry of southern Tibet: Implications for the role of mantle thickening and delamination in the Himalayan orogen. *Geology* 2005; **33:** 181−4.
16. Zachos J, Pagani M, Sloan L *et al.* Trends, rhythms, and aberrations in global climate 65 Ma to present. *Science* 2001; **292**: 686−93.
17. Huang W, Dupont-Nivet G, Lippert PC *et al.* What was the Paleogene latitude of the Lhasa terrane? A reassessment of the geochronology and paleomagnetism of Linzizong volcanic rocks (Linzhou Basin, Tibet). *Tectonics* 2015; **34**: 594−622.
18. Bowen GJ and Wilkinson B. Spatial distribution of δ^{18}O in meteoric precipitation. *Geology* 2002;

30: 315−8.
19. Westerhold T, Marwan N, Drury AJ *et al.* An astronomically dated record of Earth's climate and its predictability over the last 66 million years. *Science* 2020; **369**: 1383−7.
20. Hoke GD, Liu-Zeng J, Hren MT *et al.* Stable isotopes reveal high southeast Tibetan Plateau margin since the Paleogene. *Earth Planet Sci Lett* 2014; **394**: 270−8.
21. Farnsworth A, Lunt DJ, Robinson SA *et al.* Past East Asian monsoon evolution controlled by paleogeography, not CO_2. *Sci Adv* 2019; **5**: eaax1697.
22. Inglis GN, Bragg F, Burls NJ *et al.* Global mean surface temperature and climate sensitivity of the early Eocene Climatic Optimum (EECO), Paleocene–Eocene Thermal Maximum (PETM), and latest Paleocene. *Clim Past* 2020; **16**: 1953−68.
